# Cytokinin and ALOG proteins regulate pluripotent stem cell identity in the moss *Physcomitrium patens*

**DOI:** 10.1126/sciadv.adq6082

**Published:** 2024-08-28

**Authors:** Yuki Hata, Juri Ohtsuka, Yuji Hiwatashi, Satoshi Naramoto, Junko Kyozuka

**Affiliations:** ^1^ Graduate School of Life Sciences, Tohoku University, Sendai 980-8577, Japan.; ^2^ School of Food Industrial Sciences, Miyagi University, Sendai 982-0215, Japan.; ^3^ Department of Biological Sciences, Faculty of Science, Hokkaido University, Sapporo 060-0810, Japan.

## Abstract

The shoot apical meristem (SAM) contains pluripotent stem cells that produce all the aerial parts of the plant. Stem cells undergo asymmetric cell divisions to self-renew and to produce differentiating cells. Our research focused on unraveling the mechanisms governing the specification of these two distinct cell fates following the stem cell division. For this purpose, we used the model organism *Physcomitrium patens*, which features a singular pluripotent stem cell known as the gametophore apical cell. We show that the activity of cytokinins, critical stem cell regulators, is restricted to the gametophore apical cell due to the specific localization of PpLOG, the enzyme responsible for cytokinin activation. In turn, PpTAW, which promotes differentiating cell identity of the merophyte, is excluded from the gametophore apical cell by the action of cytokinins. We propose a cytokinin-based model for the establishment of asymmetry in the pluripotent stem cell division.

## Teaser

Cytokinin is confined to the single pluripotent stem cell and excludes ALOG proteins, the differentiation factor, in bryophytes.

## INTRODUCTION

**Fig. 1.**
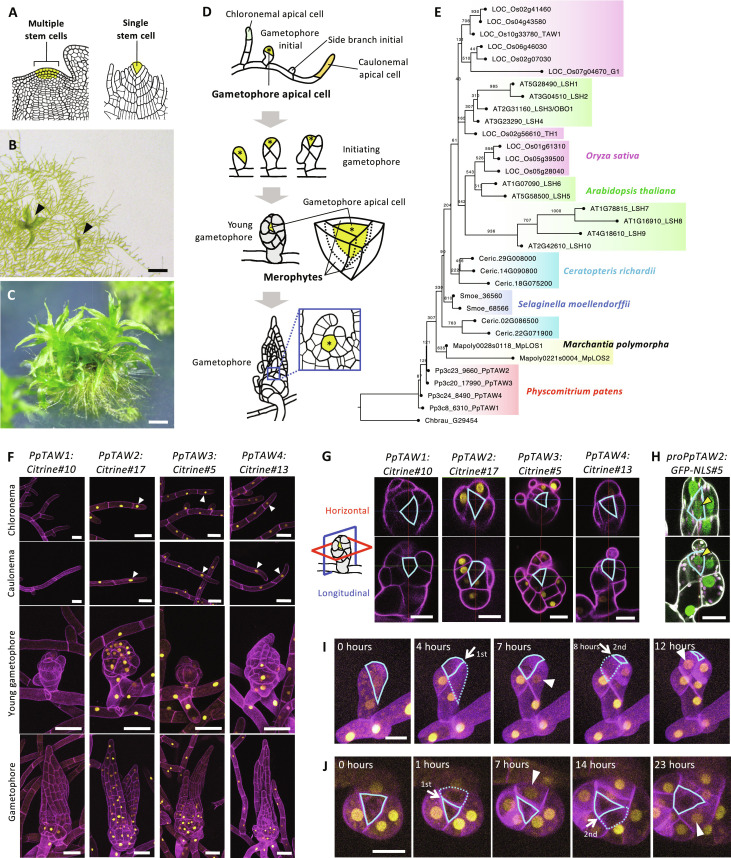
The PpTAW protein is excluded from the gametophore apical cell through posttranscriptional regulation. (**A**) Structure of SAM in land plants. Stem cells are indicated by yellow color. (**B** and **C**) Protonemata of *P. patens*, having young gametophores (B) and mature gametophores (C). Black arrowheads indicate young gametophores. (**D**) Schematic representation of gametophore initiation and development. Yellow cells with an asterisk indicate the gametophore apical cell. (**E**) Phylogenetic tree of ALOG proteins in land plants. The bootstrap value is indicated at the branch point. (**F**) Localizations of PpTAW:Citrine fluorescence (yellow) in protonema, young gametophore and gametophore. PpTAW:Citrine fluorescence is visible in the chloronemal and caulonemal apical cells (white arrowheads). (**G**) Localizations of PpTAW:Citrine fluorescence (yellow) in the SAM. The top panels show horizontal (red square) views, and the bottom panels show longitudinal (blue square) views. The outlines of the gametophore apical cell are marked with cyan lines. (**H**) Promoter activity of *PpTAW2*. GFP fluorescence (green) driven by the *PpTAW2* promoter is shown. The magenta color represents the autofluorescence of chloroplasts. Yellow arrowheads indicate the GFP fluorescence in the gametophore apical cell. (**I** and **J**) Time-lapse imaging of PpTAW2:Citrine (yellow) localization during the division of gametophore apical cells in an initiating gametophore (I) and a growing young gametophore (J). Lateral view (I) and top view (J) of growing gametophores are shown. Solid and dashed cyan lines outline the gametophore apical cell and newly formed merophyte, respectively. White arrows indicate the cell division planes formed during the observation time. White arrowheads indicate the PpTAW2:Citrine signal that appeared in the merophyte after division of the gametophore apical cell. Scale bars, 500 μm (B), 300 μm (C), 50 μm (F), and 20 μm [(G) to (J)].

All the diverse tissues and organs in multicellular organisms originate from stem cells (*1*–*3*). In plants, stem cells in the shoot apical meristem (SAM) underlie aerial shoot growth (*4*). The root apical meristem is the originator of all of the cells in the root system (*5*). The advent of stem cells that can produce distinct cell types has been the driving force behind the remarkable morphological diversity of plants, which enabled the colonization of terrestrial environments and the success of land plants (*6*–*9*). In flowering plants (angiosperms), a group of stem cells is maintained at the center of the SAM, while the SAM of bryophytes and most ferns contains a single stem cell known as the apical cell (Fig. 1A) (*10*, *11*). Studies have shown that core regulatory mechanisms controlling the function of the SAM are conserved in land plants in spite of the differences in SAM structure (*12*–*20*). Cytokinins, a class of phytohormones, have been shown to be a common factor promoting stem cell identity within the SAM in land plants (*19*). A crucial role of the stem cell is to perpetuate asymmetric cell divisions to ensure both self-renewal and the production of differentiating progenitor cells (*3*). However, the precise mechanism by which cytokinins promote the fate of the stem cells and control the asymmetric division of these cells in plants remains largely unknown.

*Physcomitrium patens* is a suitable model for studying SAM development (Fig. 1, B and C) (*21*, *22*). The SAM of *P. patens* exhibits a simple structure with clear cell types and is applicable to live imaging. The cell division pattern in the SAM, which has been well-documented, is highly coordinated (*23*). During the life cycle of *P. patens*, filaments called chloronemata grow following the germination of spores. The chloronemal apical cell then undergoes a transition to become the caulonemal apical cell. Chloronemata and caulonemata are collectively called protonemata. The growth of the protonemata is characterized by the division and extension of the chloronemal and caulonemal apical cells at their tips (Fig. 1D). Most side branch initials generated on the protonema grow as filaments, contributing to the two-dimensional expansion of the plant. Nevertheless, a subset of these side branch initials gives rise to gametophores (Fig. 1D, gametophore initial). The gametophore apical cell is a pluripotent stem cell, different from protonemal apical cells, which have limited stem cell capability for self-renewal (*21*). The gametophore apical cell follows a characteristic division pattern, resulting in the formation of a SAM featuring a central tetrahedral apical cell and an outer cell known as the merophyte. The merophyte differentiates to form a leaf and a segment of the stem (Fig. 1D, gametophore).

## RESULTS

### PpTAW proteins are excluded from the gametophore apical cell

ALOG [Arabidopsis light-dependent short hypocotyl (LSH) and *Oryza* G1] proteins are transcription factors conserved in land plants (*24*). *ALOG* genes exhibit high sequence similarity across the coding region among land plants (fig. S1). In angiosperms, *ALOG* genes are expressed in the boundary region that lies between the SAM and differentiating organ primordia. They regulate SAM indeterminacy, boundary differentiation, and lateral organ size (*25*–*29*). In the bryophyte *Marchantia polymorpha*, a single ALOG gene, *LATERAL ORGAN SUPRESSOR 1* (Mp*LOS1*), directly controls the identity of differentiating cells and, indirectly, the activity of the gametophyte apical cell (*30*).

There are four *ALOG* genes in the *P. patens* genome. We named them *PpTAW1* (Pp3c8_6310), *PpTAW2* (Pp3c23_9660), *PpTAW3* (Pp3c20_17990), and *PpTAW4* (Pp3c24_8490), after the rice *TAWAWA1* (*TAW1*) gene (Fig. 1E and fig. S1) (*26*). We first examined the localization of the four PpTAW proteins using reporter lines (*PpTAW1:Citrine#10*, *PpTAW1:Citrine#12*, *PpTAW2:Citrine#17*, *PpTAW2:Citrine#9*, *PpTAW3:Citrine#5*, *PpTAW3:Citrine#2*, *PpTAW4:Citrine#13*, *PpTAW4:Citrine#15*) in which the Citrine gene is inserted at the C terminus of the coding sequence. Consistent with the function of ALOG proteins as transcription factors, the fluorescence of the fusion protein localized in the nucleus in all lines. The signal intensity varied among the four *PpTAW*s, with the highest observed for the *PpTAW2:Citrine* fusion and the lowest for the *PpTAW1:Citrine* fusion. *PpTAW2:Citrine*, *PpTAW3:Citrine*, and *PpTAW4:Citrine* reporter lines showed fluorescence in almost all tissues, including the protonemata, side branch initials, and gametophores. *PpTAW1:Citrine* reporter lines showed weak signals at the base of gametophores (Fig. 1F and fig. S2A). We further examined the localization of the PpTAW:Citrine signal within the gametophore apical cell in the SAM. The gametophore apical cell is shaped as a regular tetrahedron; thus, it appears as a triangular shape at the center of the SAM in both the horizontal and longitudinal sections. The signal of all four PpTAW:Citrine fusion proteins was excluded from the gametophore apical cell (Fig. 1G and fig. S2B). Because we confirmed that the spatial localization patterns of PpTAW2-4 proteins are similar, we focused our subsequent analysis on *PpTAW2:Citrine*, which showed the strongest signal. To test whether the PpTAW2 protein localization is regulated at the transcriptional level, we generated *proPpTAW2:GFP-NLS* lines expressing nuclear-localized green fluorescent protein (GFP) under the control of the *PpTAW2* promoter. The GFP fluorescence was detected in the gametophore apical cell, indicating that the transcription of *PpTAW2* occurs in the gametophore apical cell (Fig. 1H and fig. S2, C and F). These results suggest that posttranscriptional regulation of *PpTAW2* expression takes place, inhibiting PpTAW2 protein accumulation in the gametophore apical cell. In contrast, PpTAW2-4 proteins were found to accumulate in the protonemal apical cells (Fig. 1F and fig. S2A). These observations suggest a correlation between the absence of the PpTAW proteins and gametophore apical cell identity.

We performed live imaging analysis of the PpTAW2:Citrine fusion protein in the gametophore apical cell during the early stages of gametophore initiation (Fig. 1I, fig. S2D, and movies S1 and S2). The weak PpTAW2:Citrine signal was observed in the gametophore apical cell at the two-cell stage (0 hours in Fig. 1I). It seems plausible that the PpTAW2:Citrine in the gametohore apical cell at the two-cell stage originated from the signal present in the caulonema cell from which the bud derived. PpTAW2:Citrine signal in the gametohore apical cell became weaker. After the division of the gametophore apical cell (4 hours in Fig. 1I), the PpTAW2:Citrine signal became visible in the merophyte cell but not in the apical cell (7 hours in Fig. 1I). This pattern was repeated in the following divisions of the gametophore apical cell. As the gametophore continued to grow, PpTAW2:Citrine fluorescence appeared only in the cell destined to become the merophyte following the division of the apical cell (Fig. 1J, fig. S2E, and movies S3 and S4). These results indicate that the PpTAW2 protein was effectively removed or absent from the gametophore apical cell from the first division of the gametophore apical cell, and the pattern of PpTAW2 localization persisted throughout gametophore development.

### Cytokinins repress PpTAW2 protein accumulation in the gametophore apical cell

**Fig. 2.**
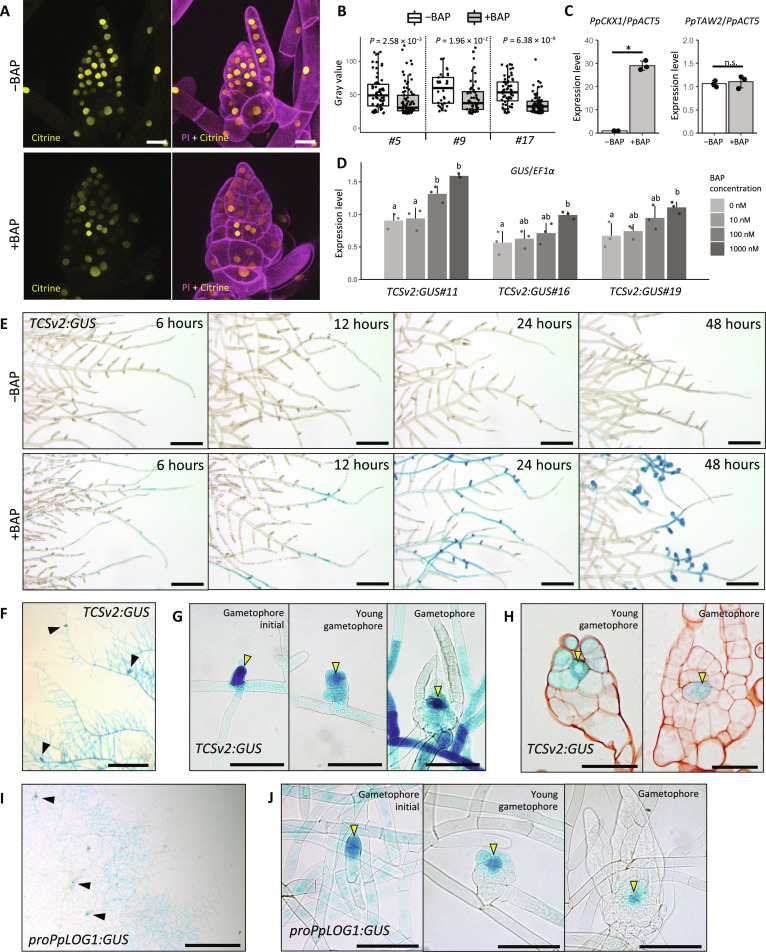
Cytokinins localize in the gametophore apical cell and repress PpTAW2 protein accumulation. (**A**) Effects of cytokinin treatment (100 nM BAP, 3 days) on the accumulation of PpTAW2:Citrine (yellow). (**B**) Signal intensity of PpTAW2:Citrine in control (−BAP) or cytokinin-treated (+BAP) gametophores in three independent lines. Plant culture condition is the same as that in (A). Citrine fluorescence in nuclei was measured by quantifying using ImageJ. Three gametophores were used in each condition. Statistical significance was examined using Student’s *t* test (*n* ≧ 33). (**C**) Effects of cytokinin (BAP) treatment on the *PpTAW2* mRNA expression. mRNA levels of *PpCKX1* and *PpTAW2* were quantified by qPCR. Plant culture condition is the same as that in (A). Statistical significance was assessed using Student’s *t* test (*n* = 3, *P < 0.05). n.s., not significant. (**D**) Response of *TCSv2:GUS* expression to the cytokinin (BAP) measured by qRT-PCR. Three independent lines (#11, #16, and #19) were examined. The BAP treatment was conducted for 3 hours. Statistical significance was evaluated by post hoc test (*n* = 3, *P* < 0.05). (**E**) GUS activity of *TCSv2:GUS* in BAP-treated (1000 nM) protonemal tissues at the several time points after starting the BAP treatment. (**F** to **H**) GUS activity of *TCSv2:GUS* in whole tissues (F), developing gametophores (H), and transversal sections of SAM in the gametophores (I). Black arrowheads in (F) indicate gametophores formed on protonema. Yellow arrowheads in (G) and (H) indicate the gametophore apical cell. (**I** and **J**) GUS activity of *proPpLOG1:GUS* in whole tissues (I) and developing gametophores (J). Black arrowheads indicate gametophores formed on protonemata. Yellow arrowheads indicate strong GUS signal observed at the gametophore apical cell. Scale bars, 20 μm (A), 50 μm (H), 100 μm [(G) and (J)], 200 μm (E), and 500 μm [(F) and (I)].

The PpTAW2 protein is present in the protonemal apical cells, which are unipotent stem cells, but is absent in the gametophore apical cell, a pluripotent stem cell. Cytokinins promote the generation of the gametophore apical cells but do not induce the generation of protonemal stem cells (*12*, *19*, *31*, *32*). These prompted us to investigate whether PpTAW2 protein accumulation is suppressed by cytokinins in the gametophore apical cell. To explore this possibility, we first treated plants expressing the PpTAW2:Citrine fusion with a cytokinin [6-benzylaminopurine (BAP)] and subsequently confirmed the induction of *PpCKX1*, an ortholog of *CYTOKININ OXIDASE 1* (*CKX1*), a cytokinin-responsive gene (*19*). The intensity of the PpTAW2:Citrine fluorescence was significantly decreased in young gametophores treated with BAP (Fig. 2, A and B), while the endogenous *PpTAW2* mRNA levels were not affected (Fig. 2C). These results support our assumption that PpTAW2 protein accumulation is repressed posttranscriptionally by cytokinins. There are several possible mechanisms for posttranscriptional repression of PpTAW2. These mechanisms may include inhibition of translation, protein degradation, and symplastic transportation from the gametophore apical cell. Investigating these mechanisms should be the next step in gaining a better understanding of how PpTAW2 expression is regulated.

We examined the spatial localization of cytokinins in the SAM using the *TWO COMPONENT SENSOR version2* (*TCSv2*) system with GUS (β-glucuronidase) as a reporter (*33*). First, we confirmed that the *GUS* expression was induced by the cytokinin treatment in a concentration-dependent manner (Fig. 2D). On the other hand, *GUS* expression was not induced by a synthetic auxin (1-naphthaleneacetic acid), kaurenoic acid, a gibberellin-related compound active in *P. patens*, indicating that the *TCSv2* specifically responds to cytokinins (fig. S3A). These results indicate that *TCSv2* is functional as a cytokinin sensor in *P. patens*. We also examined spatial and temporal pattern of *GUS* expression patterns in response to cytokinin treatment. Gametophores initiated at branching sites of protonemal tissues by 48 hours after the start of cytokinin treatment. Weak GUS signals were detected around the branching sites of protonemal tissues at 6 hours. The signals became strong at 12 hours, with the strongest signals observed at the side branch initials of the single cell stage at 24 hours. Last, strong GUS signals were observed in young gametophores at 48 hours (Fig. 2E). The localization of the GUS signal triggered by the cytokinin treatment is well coincident with the function of cytokinin in promoting gametophore apical cell formation. This indicates that cytokinin signaling occurs in a developmental context-dependent manner, and *TCSv2* is a good marker for cytokinin signaling. Next, we analyzed GUS signal of *TCSv2* lines in the normal growth condition. The *TCSv2:GUS* lines showed a weak to moderate GUS signal in most protonemal cells. In contrast, intense signals were observed in the gametophore apical cells and gametophores at the early stage (Fig. 2, F and G). As the gametophore grew, the GUS signal became restricted to its central part (Fig. 2G). We further confirmed the presence of strong GUS expression in the gametophore apical cell in the young and growing gametophore (Fig. 2H). These analyses revealed that intense cytokinin signaling occurred in the gametophore apical cell from the single-cell stage, and the cytokinin signaling was consistently maintained in the gametophore apical cell as it continued to develop.

LONELY GUY (LOG) catalyzes the final step of the cytokinin biosynthesis pathway (*34*). *P. patens* has nine genes encoding LOG orthologs (fig. S3, B and C) (*35*). To clarify the localization of cytokinin biosynthesis, we generated marker lines in which the *GUS* gene is fused with the sequence of the *PpLOG1* gene promoter, which exhibits a strong activity (*36*, *37*). Strong GUS activity was detected in the gametophore initial cell and the gametophore apical cell in the SAM, similar to the GUS signal localization observed with the *TCSv2:GUS* lines (Fig. 2, I and J). These results suggest that specific localization of *LOG* expression in the gametophore apical cell restricts cytokinin activity to the gametophore apical cell. The simultaneous localization of cytokinin signaling and PpTAW2 protein and the suppression of PpTAW2 protein accumulation by cytokinins provide a strong support to our hypothesis that PpTAW2 protein accumulation is inhibited by cytokinins in the gametophore apical cell.

### Inhibition of *PpTAW*s’ function causes overgrowth of protonemata and leaves and impairs merophyte differentiation

**Fig. 3.**
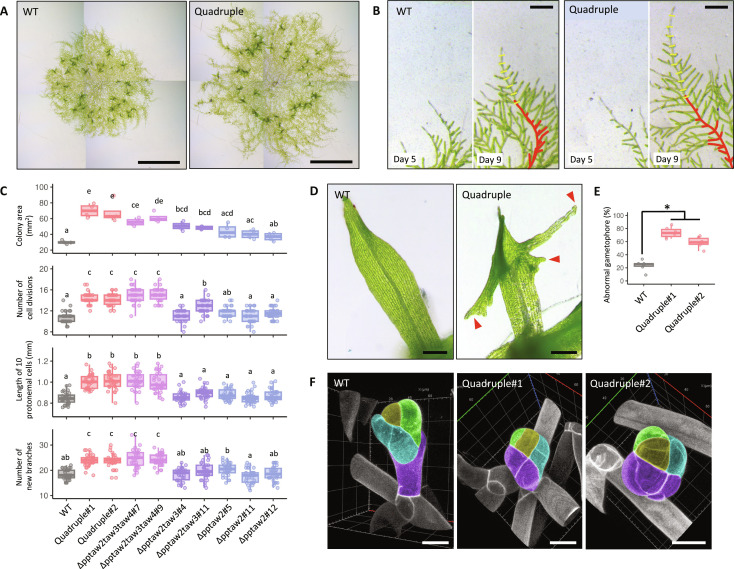
Disruption of *PpTAW*s’ function causes overgrowth of protonemata and abnormal organ differentiation. (**A**) Colonies of wild type (WT) and *PpTAW* quadruple mutant at 23 days. (**B**) Protonemata of WT and the quadruple mutant from days 5 to 9 after inoculation. Red lines outline the silhouettes of day 5 plants. Yellow lines indicate the position of newly formed cell wall by division of the caulonemal apical cell. (**C**) Quantification of colony growth of WT and *PpTAW* loss-of-function mutants. Colony area of day 23 plants (*n* = 4), number of caulonemal apical cell divisions between days 5 and 9 after inoculation (*n* = 30), length of caulonemal cells (*n* = 30), and number of new branches formed on a caulonemal filament (*n* = 30). Statistical significance was evaluated using the post hoc test (*P* < 0.05). (**D**) Leaves in gametophores of WT and quadruple mutants. Red arrowheads indicate abnormal elongation of leaf margins. (**E**) Frequency of gametophores showing abnormal leaf elongation in WT and quadruple mutants. Statistical significance was examined by Student’s *t* test (*n* = 6, *P < 0.05). (**F**) Initiating gametophores in WT and quadruple mutants. The gametophore apical cell is shown in yellow. Green-, magenta-, and cyan-colored cells are progenitor cells derived from different merophytes. Scale bars, 4 mm (A), 200 μm [(B) and (D)], and 20 μm (F).

To reveal the function of *PpTAW*s, we generated loss-of-function mutants by homologous recombination. Double mutants of *PpTAW2* and *PpTAW3* (Δ*pptaw2*Δ*pptaw3*) showed a significant increase in colony size. The increase in colony size was intensified in lines bearing mutations in multiple *PpTAW* genes, and it reached its highest level in the quadruple mutants (Fig. 3, A to C, and fig. S4A). The defect was caused by an increase in both the number of cell divisions and the length of the protonemal cells. Colony growth in *P. patens* exclusively depends on the tip growth and division of protonemal apical cells (*21*). Thus, PpTAWs inhibit the activity of protonemal apical cells to elongate and divide.

Continuous observation of the same plants revealed that the number of protonemal branches generated between days 5 and 9 was higher in the quadruple mutants than that in wild type (WT) (Fig. 3, B and C). These results indicate that the four *PpTAW* genes redundantly function in repressing division and growth of protonemal apical cells and generation of side branch initials.

Gametophore development was also affected in the quadruple mutants. Leaves, which originate from the merophyte, undergo coordinated cell divisions and growth to take a flat and elliptical shape (*38*). In the quadruple mutants, the leaves frequently exhibited excessive growth (Fig. 3, D and E). These observations suggest that *PpTAW*s play a crucial role in regulating the timing of cell growth and divisions. The rhizoid, a filamentous tissue at the base of the gametophore, gradually becomes brown as it matures (*39*). The browning of rhizoid cells is inhibited in the quadruple mutants, indicating that the *PpTAW*s genes are also required to promote rhizoid cell maturation (fig. S4, B and C).

**Fig. 4.**
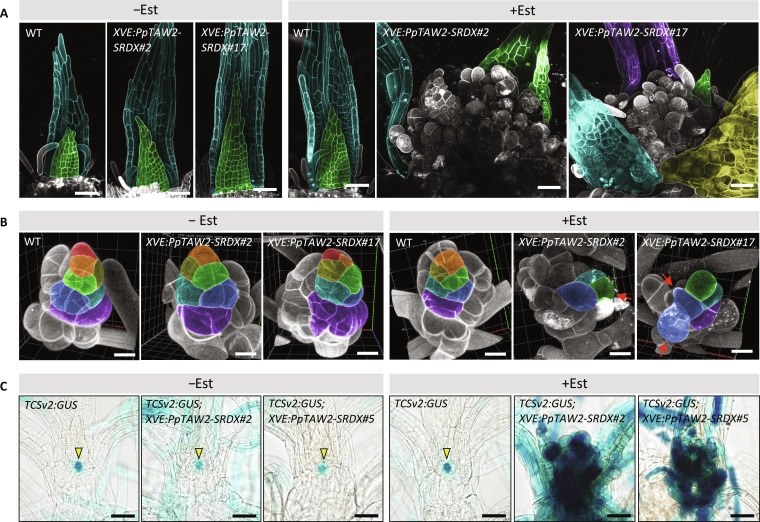
Dominant repression of transcriptional activity of PpTAWs disturbs the merophyte identity. (**A**) The gametophore apex in WT and *XVE:PpTAW2-SRDX* lines after β-estradiol application (0 or 100 nM, 4 weeks). Green-, cyan-, magenta-, and yellow-colored tissues are leaf primordia or young leaves. (**B**) Cell division patterns of merophytes in WT and *XVE:PpTAW2-SRDX* lines after application of 100 nM β-estradiol (+Est) or solvent (−Est) for 1 week. Clonal sectors derived from the same merophyte are shown using the same color. Red arrows indicate irregular cell elongation. (**C**) Localization of GUS activity of *TCSv2:GUS* reporter in WT or *XVE:PpTAW2-SRDX* background after application of 100 nM β-estradiol (+Est) or solvent (−Est) for 1 week. Yellow arrowheads indicate gametophore apical cells. Scale bars, 20 μm (B) and 50 μm [(A) and (C)].

We did not observe morphological alterations in the SAM of gametophores in the quadruple mutants even in the later stage of development (Fig. 3F and fig. S4D). To further investigate the role of PpTAWs as transcription regulators in SAM development, we generated β-estradiol–inducible lines expressing *PpTAW2-SRDX*. Because the SRDX system leads to strong dominant repression of target genes (*40*), PpTAW2-SRDX dominantly disturbs the normal function of PpTAWs to regulate downstream genes. In these lines, the expression of PpTAW2-SRDX is ubiquitously induced by β-estradiol application (*41*). Induction of *PpTAW2-SRDX* during gametophore development caused the proliferation of irregular cell masses at the top of the gametophore (Fig. 4A). Continuous observation of leaf development in these plants revealed that the growth pattern of cells in the leaf primordia was severely disturbed. However, the gametophore apical cell looked almost normal in *PpTAW2-SRDX* lines (Fig. 4B). We used *TCSv2:GUS* to clarify the identity of the irregular cells. GUS signal was specifically localized to gametophore apical cells in control plants, whereas the GUS signal was observed in entire parts of irregular cell masses formed by *PpTAW2-SRDX* induction (Fig. 4C). These results indicate that the merophyte fails to establish its identity. ALOG proteins function both as transcriptional activators and repressors of downstream genes (*42*–*44*). If PpTAWs also act as both activators and repressors, then disturbance of PpTAWs’ function in the SRDX system should result in pronounced disorders in gametophore development. On the other hand, the loss-of-function mutants have no significant phenotype in the merophyte development. These results suggest that the downstream genes of PpTAWs are important for merophyte identity and regulated redundantly by other unknown factors. However, we cannot rule out the possibility that abrupt inhibition of PpTAWs’ function caused the disturbance of gametophore development as it requires strict spatiotemporal regulation of gene expression. Further analysis of PpTAWs molecular function is necessary to distinguish between these two possibilities. In summary, the analysis of the quadruple mutants and *PpTAW2-SRDX* overexpression lines suggests that PpTAWs suppress the tip growth and division of the protonemal apical cells and formation of side branch initials while promoting the establishment of merophyte identity.

### Ectopic overexpression of PpTAW2 suppresses protonemal growth and stem cell initiation

**Fig. 5.**
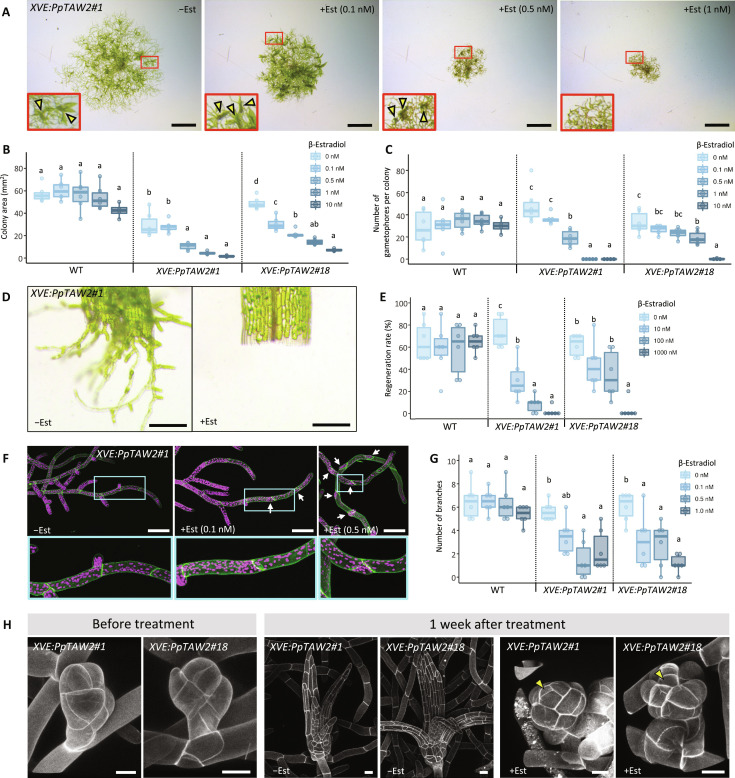
Ectopic overexpression of PpTAW2 inhibits protonemal apical cell activity and gametophore initiation. (**A**) Colonies of *XVE:PpTAW2* plants grown with (+Est) or without (−Est) β-estradiol for 21 days. A close-up view of the framed area is shown at the lower left corner. Yellow arrowheads indicate gametophores. (**B**) Colony area of WT and *XVE:PpTAW2* plants grown with different concentrations of β-estradiol for 21 days. (**C**) Number of gametophores of WT and *XVE:PpTAW2* plants grown with different concentrations of β-estradiol for 21 days. (**D**) Protonema regeneration from detached leaves of *XVE:PpTAW2* plants treated with 1 μM β-estradiol (+Est) or without β-estradiol (−Est), and grown for 3 days. (**E**) Frequency of protonemal regeneration from detached leaves in WT and *XVE:PpTAW2* lines 3 days after treatment with different concentrations of β-estradiol. (**F**) Confocal images of the protonemata of *XVE:PpTAW2* plants treated with (+Est) or without (−Est) β-estradiol. The concentration of β-estradiol is shown in each top subpanel. Close-up views of the cyan framed areas in the top panels are shown in the bottom panels. White arrows indicate sites where the growth of side branch initials is arrested. Magenta color is the autofluorescence of chloroplasts. (**G**) Quantification of the suppression of side branch initial elongation observed from the ninth caulonemal cells to the tip of the gametophore of WT and *XVE:PpTAW2* lines grown with different concentration of β-estradiol. (**H**) Confocal images of the developing gametophores of *XVE:PpTAW2* lines before or after β-estradiol application (0 or 100 nM, 1 week). Yellow arrowheads indicate gametophore apical cells. Statistical significance was assessed using the post hoc test (*P* < 0.05, *n* = 4 to 6) [(B), (D), (F), and (G)]. Scale bars, 2 mm (A), 200 μm (D), 100 μm (F), and 20 μm (H).

We further analyzed the function of PpTAWs and the significance of their proper spatial localization using PpTAW2 inducible lines, *XVE:PpTAW2*, in which PpTAW2 can be induced by application of β-estradiol (*41*). We confirmed the presence of PpTAW2 in all tissues including the gametophore apical cell following β-estradiol treatment by using a *XVE:PpTAW2*-*Citrine* line (fig. S5). Induction of XVE:PpTAW2 by β-estradiol application resulted in plant growth inhibition (Fig. 5, A to C). Colony size reduction was observed in a β-estradiol concentration-dependent manner, suggesting an inhibitory effect of PpTAW2 on protonemal apical cell activity (Fig. 5, A and B). Differentiated tissues of *P. patens* retain the capacity to form new protonemal apical cells and to regenerate protonemata (*45*). We also examined the effects of PpTAW2 overexpression on protonemal apical cell regeneration. *XVE:PpTAW2* gametophores were cultured in the presence of β-estradiol, and then leaves were cut and cultured on the medium without β-estradiol. The formation of protonemal apical cells from the section of the cut leaves was suppressed in plants treated with β-estradiol in a concentration-dependent manner (Fig. 5, D and E). This provides further support for the inhibitory role of PpTAW2 in specifying protonemal stem cell identity and activity. In addition, applying a higher concentration (0.5 nM) of β-estradiol suppressed the formation of side branch initials (Fig. 5, F and G). Gametophore formation was almost fully suppressed by applying 1 nM β-estradiol, suggesting that PpTAWs inhibit the specification of gametophore apical cell identity and/or its activity (Fig. 5, A and C). We also examined the effects of PpTAW induction on initiating gametophore buds. Application of β-estradiol to initiating gametophores caused severe suppression of leaf growth (Fig. 5H). The observed phenotypes suggest that leaf primordia development was prematurely arrested due to promoting cell differentiation (*46*). These results support our hypothesis that PpTAW function is to promote cell differentiation.

## DISCUSSION

**Fig. 6.**
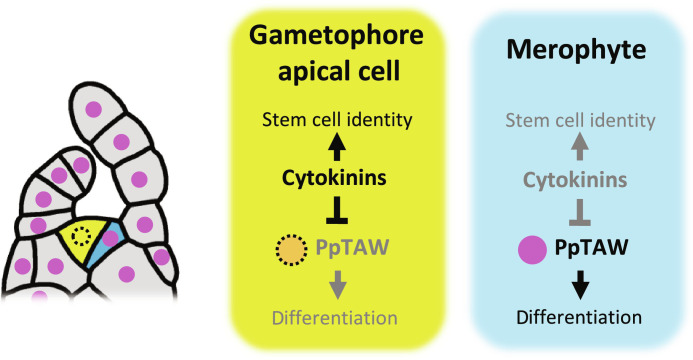
Model for the establishment of asymmetric cell fates of the gametophore apical cell and the merophyte. Cytokinin levels are elevated in the gametophore apical cell where it promotes pluripotent stem cell identity and represses PpTAW protein accumulation. On the other hand, cytokinin levels decrease in the merophyte after cell division, allowing accumulation of PpTAW. PpTAW promotes merophyte identity and facilitates merophyte differentiation. Therefore, the gametophore apical cell–specific accumulation of cytokinins and the suppression of PpTAW accumulation by cytokinins are essential for establishing the asymmetry after the cell division of the gametophore apical cell.

We showed that cytokinin activity is localized to the gametophore apical cell in the SAM of *P. patens*. Conversely, PpTAW promotes merophyte identity and is excluded from the gametophore apical cell due to cytokinin activity. On the basis of these findings, we propose a model to describe how the SAM of *P. patens*, which contains a single pluripotent stem cell known as the gametophore apical cell, is initiated and maintained (Fig. 6). Cytokinins work as pluripotent stem cell factors, while PpTAW is a differentiation factor that promotes the merophyte identity. In addition, cytokinins suppress the accumulation of PpTAW proteins. Cytokinin activity is restricted to the gametophore apical cell as consequence of the localization of LOG, the final enzyme of the cytokinin biosynthesis pathway, within this cell, resulting in PpTAW being specifically excluded from the gametophore apical cell. This process leads to the autonomous establishment of asymmetric cell identity, pluripotent stem cell activity of the gametophore apical cell, and differentiating cell activity of the merophyte following the division of the gametophore apical cell.

This is the first identification of the mechanism for the establishment of asymmetric cell fate in bryophyte SAM. However, several questions remain unanswered. First, the absence of significant phenotypes in merophyte development in *PpTAW* loss-of-function mutants suggests the presence of redundant yet unidentified pathways involved in merophyte differentiation. Second, the mechanisms of posttranscriptional repression of PpTAW accumulation by cytokinin and the other factors promoting this repression remain largely unknown. Cytokinin not only is specifically localized to the gametophore apical cell in the gametophores but also is present in differentiated protonemal tissues where PpTAW accumulates. This suggests the presence of additional unidentified factors, beside cytokinn, that repress PpTAW accumulation in the gametophore apical cell. Third, the exact role of cytokinin in promoting apical cell formation remains unclear. While it is established that cytokinin promotes gametophore apical cells, cytokinin treatment is sufficient to promote the initiation of gametophore apical cells in the side branch initial cells of the protonema and on the surface of the gametophore stem, but not in other tissues (*19*, *47*). In this study, we demonstrated that cytokinin application to the *TCSv2:GUS* reporter resulted in a markedly biased response toward the side branch initial cells in protonemata, indicating that cells exhibiting strong cytokinin responsive are already specified. Furthermore, the loss-of-function mutants of *PpCHK*s, cytokinin receptors, still form gametophore apical cells (*19*, *47*). These findings suggest the existence of additional factors involved in the specification and establishment of pluripotent stem cell identity in addition to cytokinin. Last, the mechanisms underlying the gametophore apical cell–specific expression of *PpLOG1*, likely a key factor for the confinement of cytokinin activity to the gametophore apical cell, are also unknown. The next critical step in understanding the nature of the pluripotent stem cell is the elucidation of the mechanisms underlying the specific localization of *PpLOG*.

*LOG* genes are expressed in the SAM, which contains the stem cell zone of angiosperms (*34*, *48*). Our finding that *PpLOG1* expression is confined to the gametophore apical cell indicates commonality of the mechanism controlling the SAM despite the difference in the number of stem cells present within the SAM. This study suggests a deep homology among plant pluripotent stem cells in land plants. Acquiring the ability to amplify the stem cells might have been crucial for the evolution of angiosperm SAM. Considering that *LOG* is expressed in multiple cells at the top of the SAM and promotes stem cell activity in angiosperms, increasing the size of the *LOG* expressing region might have been critical for the evolution of the SAM. In angiosperms, cytokinins activate the expression of *WUSCHEL* (*WUS*), a master regulator of stem cell identity within the SAM (*49*). Specifically, *WUS* regulates the number of stem cells in the SAM in angiosperms (*49*). At the same time, cytokinin biosynthesis and class I KNOTTED LIKE HOMEOBOX (KNOXI) family transcription factors form a positive feedback loop, contributing to the maintenance of the undifferentiated state of SAM (*50*). However, recent studies suggest that the function of WUS in the SAM has evolved after the divergence of seed plant lineages (*17*, *51*–*53*). The cytokinin-KNOXI interaction is conserved in sporophytic meristem but not in gametophyteic SAM in *P. patens* (*50*, *54*). Therefore, acquisition of additional factors promoting stem cell identity, including WUS and KNOXI, might also have been necessary for the evolution of a sporophytic SAM containing multiple stem cells.

ALOG genes are important for the indeterminacy of the inflorescence meristem and lateral organ development in angiosperms (*25**–**29*). A recent study revealed that an ALOG gene in *M. polymorpha* represses lateral organ growth and maintains SAM activity through non–cell-autonomous mechanisms (*30*). Because lateral organs evolved independently in angiosperms and bryophytes, it was suggested that the ALOG genes are involved in the convergent evolution of lateral organ development (*30*). Our results also indicate a common function of ALOG genes in regulating lateral organ development in *P. patens*. However, the function of the ALOG genes in the maintenance of SAM activity was not supported in *P. patens*, suggesting diversification of ALOG gene function in different bryophyte lineages. Given that the lateral organs of liverworts and mosses have independent origins (*11*), this diversification of ALOG gene function might reflect the differences in their evolutionary origins. Comparing the functions and downstream factors of ALOG genes across different land plant lineages would be interesting for understanding the common molecular mechanisms underlying the convergent evolution of shoot structure in land plants.

## MATERIALS AND METHODS

### Plant materials and culture conditions

The Gransden Wood strain (1962) of *P. patens* was used as WT (*55*). *P. patens* plants were cultured in BCDAT medium, under continuous light at 25°C, as described by Nishiyama *et al*. (*56*), and then transferred to media for analysis.

For the observation of plant colonies of the *PpTAW*s multiple mutants, *PpTAW:Citrine* reporter lines, *proPpTAW2:GFP-NLS* reporter lines, *TCSv2:GUS* reporter lines, and *proPpLOG1:GUS* reporter lines, small amounts of protonemal tissues were cultured between two cellophane membranes (PL#300, Futamura Chemical Co. Ltd.) on BCD medium from 2 to 3 weeks. In case of cytokinin treatment of *PpTAW2:Citrine* reporter lines, plant tissues were cultured in liquid BCD medium containing 100 nM BAP (Sigma-Aldrich) or the same amount of solvent [dimethyl sulfoxide (DMSO)] for 3 days following the BCD solid medium culture. In case of hormonal treatment of *TCSv2:GUS* reporter lines, plant tissues were cultured in liquid BCD medium containing appropriate concentration of phytohormone or the same amount of solvent following the BCD solid medium culture. BAP, 1-naphthaleneacetic acid (Sigma-Aldrich), or kaurenoic acid (Sigma-Aldrich) was used as phytohormone. For the observation of gametophores of the *PpTAW* quadruple mutants, plants were cultured on BCDAT medium for 1 month. For live imaging of *PpTAW2:Citrine;LTI6b:RFP* lines, small amounts of protonemal tissues were placed on thin BCD medium covering the bottom of a glass dish (D11140H, Matsunami Glass Ind. Ltd.) and then covered with a cellophane membrane so that plants grow in the narrow space between the glass bottom and the cellophane membrane. Gametophore production was usually observed from days 10 to 18, and gametophores in optimal developmental stage were selected and used for live imaging.

For the analysis of *XVE:PpTAW2* plants, the plants were grown on the BCD medium containing appropriate concentration of β-estradiol (Fujifilm Wako Pure Chemical Corp.) or the same amount of solvent (DMSO) as a control. Growth conditions were modified depending on the developmental stages to be analyzed. For the observation of plant growth phenotypes, plants were cultured for 3 weeks between two cellophane membranes. For the regeneration assay from detached leaves, gametophores grown on BCDAT medium for 3 weeks were collected and soaked in BCD liquid medium containing the appropriate concentration of β-estradiol or the same amount of solvent (DMSO) for 24 hours. Soaked gametophores were thoroughly washed with sterile water, and the five youngest leaves were cut from each gametophore. Detached leaves were pooled and mixed. Ten leaves were randomly selected and placed on BCDAT medium. Cellophane membranes were placed on top of the leaves, and they were cultured for 3 days. For the obsevation of gametophore development, small amounts of protonemal tissues were cultured between two cellophane membranes on BCD solid medium for 3 weeks and then transferred to BCD liquid medium containing 100 nM β-estradiol or the same amount of solvent (DMSO). The plants were further cultured for 1 week.

For the observation of PpTAW2-Citrine localization in *XVE:PpTAW2-Citrine* plants, the plants were cultured for 3 weeks between two cellophane membranes and then cultured in sterilized water containing 100 nM β-estradiol or the same amount of solvent (DMSO) for 24 hours.

For analyses of the phenotypes of *XVE:PpTAW2-SRDX* plants, first, young gametophores were produced by culture on plain BCD medium plates for 3 weeks, followed by addition of 20 ml of sterilized water containing 100 nM β-estradiol or the same amount of solvent, and an additional 4 weeks of culture. For the observation of merophyte development and *TCSv2:GUS* reporter expression, small amounts of protonemal tissues were cultured between two cellophane membranes on BCD medium for 3 to 4 weeks and then transferred to BCD medium plates containing 100 nM β-estradiol or the same amount of solvent (DMSO). The plants were further cultured for 1 week.

### Phylogenetic analysis

BlastP searches were performed on Phytozome (https://phytozome.jgi.doe.gov/pz/portal.html) using default parameter settings to look for ALOG family proteins and LOG proteins in *Oryza sativa*, *Arabidopsis thaliana*, *Ceratopteris richardii*, *Selaginella moellendorffii*, *M. polymorpha*, *P. patens*, and *Chara braunii*. For the ALOG family proteins, the amino acid sequence of TAW1 from *O. sativa* was used as query in search were aligned using Clustal Omega (www.ebi.ac.uk/Tools/msa/clustalo/), and the ALOG domain region without gaps was extracted using the Geneious software to get the multiple sequence alignments for the phylogenetic analysis. For the LOG proteins, the amino acid sequence of LOG from *O. sativa* was used as a query in the BlastP search. Amino acid sequences found in the BlastP search were aligned with Clustal Omega, and the lysine decarboxylase domain region without gaps was extracted using the Geneious software. The phylogenetic analysis was performed on PhyML (www.atgc-montpellier.fr/phyml/), based on the maximum likelihood method with 1000 times bootstrap.

### Vector construction

The pCit-aphIV vector (gift from M. Hasebe, National Institute for Basic Biology) was used to make *PpTAW:Citrine* knock-in constructs for visualization of PpTAW protein localizations. Both DNA fragments around 1.2 kilo–base pair (kbp) upstream and downstream of the stop codon of each *PpTAW* were amplified by polymerase chain reaction (PCR). The DNA fragment of the upstream region was cloned into the Eco RV site just before the Citrine coding region in the pCit-aphIV vector so that the reading frame of *PpTAW* is in frame with the Citrine’s using a linker sequence (GGAGGAGGATCA). The DNA fragment of the downstream region was cloned into the Sma I site just after the selective marker gene cassette of the pCit-aphIV.

The pPIG1b:NGGII vector (accession number AB537478) was used to make the *proPpTAW2:GFP-NLS*, *TCSv2:GUS*, and *proPpLOG1:GUS* constructs (*57*). For the *proPpTAW2:GFP-NLS* and *proPpLOG1:GUS* constructs, DNA fragments including 3.5 kbp around the promoter region of *PpTAW2* and *PpLOG1* were amplified by PCR and cloned into the Sma I site of the pPIG1b:NGGII vector using the SLiCE method, respectively (*58*). For the *TCSv2:GUS* construct, DNA fragments containing the *TCSv2* promoter were amplified using the TCSv2:3xVENUS vector as a template and cloned into the Sma I site of pPIG1b:NGGII (*33*).

pTN186 (Addgene, plasmid no. 34890), pTN182 (Addgene, plasmid no. 34888), p35S-loxP-Zeo (AB540628), and p35S-loxP-BSD (AB537973) were used to make constructs for the disruption of *PpTAW2*, *PpTAW3*, *PpTAW4*, and *PpTAW1* respectively (*13*, *54*). DNA fragments 1.2 kbp upstream and downstream of the coding region of each *PpTAW* were amplified by PCR. The DNA fragment of the upstream region was cloned into the Eco RV site before the selective marker gene cassette in the vector. The DNA fragment of the downstream region was cloned into the Sma I site after the selective marker gene cassette in the vector.

The pPGX8 vector (AB537482) was used to make *XVE:PpTAW2*, *XVE:PpTAW2-Citrine*, and *XVE:PpTAW2-SRDX* constructs (*41*). A DNA fragments containing the coding sequence of *PpTAW2* or *PpTAW2-Citrine* were amplified by PCR using genomic DNA of *P. patens* or PpTAW2:Citrine vector as template, respectively. Amplified DNA fragments were subcloned into pENTR/D-TOPO vector (Invitrogen). The sequence of *PpTAW2* or *PpTAW2-Citrine* was transferred to pPGX8 by LR reaction to generate *XVE:PpTAW2* or *XVE:PpTAW2-Citrine* construct. For the construction of *XVE:PpTAW2-SRDX*, the SRDX sequence (CTGGATCTGGATCTGGAACTGCGCCTGGGCTTTGCG) was introduced just before the stop codon of *PpTAW2* subcloned in pENTR/D-TOPO, by site-directed mutagenesis using inverse PCR (*40*). The sequence of *PpTAW2-SRDX* in pENTR/D-TOPO was transferred to pPGX8 by LR reaction to generate the *XVE:PpTAW2-SRDX* constructs. The list of primers used for vector construction is shown in table S1.

### Transformation and genotyping

Six- to 7-day-old protonema tissue cultured on BCDAT medium overlaid with cellophane was used for the transformation. Polyethylene glycol–mediated transformation was performed as described by Nishiyama *et al*. (*56*). Plasmids used for the transformation were extracted using the Fast gene plasmid mini kit (Nippon Genetics Co. Ltd.). For introduction of *PpTAW:Citrine*, *proPpTAW2:GFP-NLS*, *TCSv2:GUS*, *proPpLOG1:GUS*, *XVE:PpTAW2*, *XVE:PpTAW2-Citrine*, and *XVE:PpTAW2-SRDX* constructs, the plasmids were digested by the appropriate restriction enzymes to isolate the DNA fragments to be integrated into the genome by homologous recombination. For introduction of *PpTAW* gene disruption constructs, DNA fragments to be integrated into the genome by homologous recombination were amplified by PCR using the plasmids as template. Plasmid (15 to 20 μg) purified by ethanol precipitation was used for the transformation. After selection on the medium containing antibiotics, genomic DNA was extracted from each regenerated plant colony, and PCR amplification of the regions inside and outside of the introduced constructs was performed to confirm the integration of the construct in the genome (figs. S6 and S7). The PCR products were checked by agarose gel electrophoresis, and lanes showing bands of appropriate length were selected. For the introduction of the *LTI6b:RFP* construct, regenerated plants showing red fluorescent protein (RFP) signal at the plasma membrane were manually selected under the fluorescence stereo microscope (M165FC, Leica). The list of primers used for genotyping is shown in table S1. At least three independent knock-in lines were analyzed for each *PpTAW* gene, and lines showing representative expression patterns were selected for further analysis. Three independent *TCSv2:GUS* marker lines showing similar GUS expression patterns were selected from nine lines and used to analyze the spatial localization of GUS expression. Three independent *proPpLOG1:GUS* lines showing similar expression patterns of GUS were selected from nine lines and analyzed.

### Histochemical GUS activity assay

GUS staining was conducted following Aoyama *et al*. (*13*) with minor modifications. Plant tissues were fixed with fixation solution [0.2% (w/v) MES (pH 5.6), 0.3% (v/v) formalin, and 0.3 M mannitol] at room temperature for 10 min. After washing with 50 mM NaH_2_PO_4_ (pH 7.0), the fixed tissues were vacuum-infiltrated with a substrate solution [50 mM NaH_2_PO_4_ (pH 7.0), 0.5 mM 5-bromo-4-chloro-3-indolyl β-d-glucuronide (X-Gluc), 0.5 mM K_3_Fe(CN)_6_, 0.5 mM K_4_Fe(CN)_6_, and 0.05% (v/v) Triton X-100] for 30 min and stained at 37°C. After the staining, the tissues were fixed with 5% (v/v) formalin for 10 min and then soaked in 5% (v/v) acetic acid for 10 min. The stained and fixed tissues were dehydrated with ethanol series. The tissues were cleared by incubation in chloral hydrate solution [66% (w/w) chloral hydrate and 8% (w/w) glycerol] at 4°C overnight before imaging.

### Plant embedding and sectioning

GUS-stained plant tissues (dehydrated with ethanol series) were embedded in Technovit 7100 resin (Heraeus Kulzer) following the manufacturer’s instructions. Embedded samples were sectioned on a rotary microtome with 7-μm thickness. The obtained sections were treated with neutral red dyes as a counterstain. Multi-Mount 480 solution (Matsunami Glass Ind. Ltd.) was used as mounting agents on the slides.

### Microscopy

PpTAW:Citrine fluorescence was observed using a confocal scanning microscope (LSM880, Zeiss). Plan-Apochromat 20×/0.80 M27 objective or LD LCI Plan-Apochromat 40×/1.2 Imm Korr DIC M27 water objective was used. Cell outlines were visualized by propidium iodide (PI; 50 mg/ml) or LTI6b:RFP, a plasmamembrane marker. LTI6b:RFP was used for the live imaging. Citrine was excited at 514 nm, whereas RFP or cell walls stained with PI were excited at 543 nm. Morphology of *XVE:PpTAW2* plants, *pptaw*s loss-of-function mutants, and *XVE:PpTAW2-SRDX* plants was observed using a stereo microscope (M165FC, Leica) or the confocal scanning microscope (LSM880, Zeiss). For the confocal microscope observations, cell walls stained by PI (50 mg/ml) and chloroplasts’ autofluorescence were excited at 543 and 633 nm, respectively. The three-dimensional reconstruction of *z*-stack images was done with the ZEN black software (Zeiss). GUS-stained samples were observed with the stereo microscope (M165FC, Leica) or a light microscope (BX51, Olympus) equipped with an Olympus DP71. UPlanFl 40× objective was used.

### Quantitative RT-PCR

Collected plant samples were frozen in liquid nitrogen and crushed to a fine powder using a multi beads homogenizer (Yasui Kikai). Total RNA was extracted from the tissue powder using the NucleoSpin RNA Plant kit (Macherey-Nagel) following the manufacturer’s instructions. cDNA was synthesized by using the SuperScript VILO cDNA Synthesis Kit (Invitrogen). Real time PCR was performed with KOD SYBR (TOYOBO) on LightCycler480II (Roche). *PpEF1*α (Pp3c2_10310) or *PpACT5* (Pp3c10_17070) were used as reference genes. The list of primers used for quantitative reverse transcription (qRT)–PCR is shown in table S1.

### Data analyses and statistics

Quantitative analysis of images was performed using ImageJ software, and statistical analyses were conducted in Excel or R software. For the quantification of the PpTAW2:Citrine signal in the nuclei, nuclei regions were extracted using the Analyze Particle function, after conversion to grayscale. Mean gray value in the nuclei regions was measured. For quantification of the pigmentation in rhizoid, rhizoid cell regions and outside regions surrounding rhizoid cell were manually selected, and differences of mean gray value between cell regions and outside regions were calculated. The comparison of the two groups was conducted on Excel, and *P* values were calculated by Student’s *t* test. The comparison of more than two groups was conducted on R, and *P* values were calculated by performing the post hoc test using the multcomp package. All raw data values are presented in data S1. All experiments were repeated at least three times.

## References

[1] R. Heidstra, S. Sabatini, Plant and animal stem cells: Similar yet different. Nat. Rev. Mol. Cell Biol. 15, 301–312 (2014). 24755933 10.1038/nrm3790

[2] C. Gaillochet, J. U. Lohmann, The never-ending story: From pluripotency to plant developmental plasticity. Development 142, 2237–2249 (2015). 26130755 10.1242/dev.117614 PMC4510588

[3] L. J. Pillitteri, X. Guo, J. Dong, Asymmetric cell division in plants: Mechanisms of symmetry breaking and cell fate determination. Cell. Mol. Life Sci. 73, 4213–4229 (2016). 27286799 10.1007/s00018-016-2290-2 PMC5522748

[4] M. Lenhard, T. Laux, Shoot meristem formation and maintenance. Curr. Opin. Plant Biol. 2, 44–50 (1999). 10047572 10.1016/s1369-5266(99)80009-0

[5] P. Doerner, Root development: Quiescent center not so mute after all. Curr. Biol. 8, R42–R44 (1998). 9427641 10.1016/s0960-9822(98)70030-2

[6] L. E. Graham, M. E. Cook, J. S. Busse, The origin of plants: Body plan changes contributing to a major evolutionary radiation. Proc. Natl. Acad. Sci. U.S.A. 97, 4535–4540 (2000). 10781058 10.1073/pnas.97.9.4535 PMC34322

[7] K. J. Niklas, The evolution of plant body plans—A biomechanical perspective. Ann. of Bot. 85, 411–438 (2000).

[8] L. A. Moody, The 2D to 3D growth transition in the moss *Physcomitrella patens*. Curr. Opin. Plant Biol. 47, 88–95 (2019). 30399606 10.1016/j.pbi.2018.10.001

[9] L. A. Moody, Unravelling 3D growth in the moss *Physcomitrium patens*. Essays Biochem. 66, 769–779 (2022). 36342774 10.1042/EBC20220048 PMC9750851

[10] A. R. G. Plackett, V. S. Di Stilio, J. A. Langdale, Ferns: The missing link in shoot evolution and development. Front. Plant Sci. 6, 972 (2015). 26594222 10.3389/fpls.2015.00972 PMC4635223

[11] C. J. Harrison, Development and genetics in the evolution of land plant body plans. Philos. Trans. R. Soc. Lond. B Biol. Sci. 372, 20150490 (2017). 27994131 10.1098/rstb.2015.0490 PMC5182422

[12] N. W. Ashton, N. Grimsley, D. J. Cove, Analysis of gametophytic development in the moss, *Physcomitrella patens*, using auxin and cytokinin resistant mutants. Planta 144, 427–435 (1979). 24407386 10.1007/BF00380118

[13] T. Aoyama, Y. Hiwatashi, M. Shigyo, R. Kofuji, M. Kubo, M. Itô, M. Hasebe, AP2-type transcription factors determine stem cell identity in the moss *Physcomitrella patens*. Development 139, 3120–3129 (2012). 22833122 10.1242/dev.076091

[14] K. Von Schwartzenberg, A.-C. Lindner, N. Gruhn, J. Šimura, O. Novák, M. Strnad, M. Gonneau, F. Nogué, A. Heyl, CHASE domain-containing receptors play an essential role in the cytokinin response of the moss *Physcomitrella patens*. J. Exp. Bot. 67, 667–679 (2015). 26596764 10.1093/jxb/erv479 PMC4737067

[15] C. D. Whitewoods, J. Cammarata, Z. N. Venza, S. Sang, A. D. Crook, T. Aoyama, X. Y. Wang, M. Waller, Y. Kamisugi, A. C. Cuming, P. Szövényi, Z. L. Nimchuk, A. Roeder, M. J. Scanlon, C. J. Harrison, CLAVATA was a genetic novelty for the morphological innovation of 3D growth in land plants. Curr. Biol. 28, 2365–2376.e5 (2018). 30033333 10.1016/j.cub.2018.05.068 PMC6089843

[16] Y. Hirakawa, N. Uchida, Y. L. Yamaguchi, R. Tabata, S. Ishida, K. Ishizaki, R. Nishihama, T. Kohchi, S. Sawa, J. L. Bowman, Control of proliferation in the haploid meristem by CLE peptide signaling in *Marchantia polymorpha*. PLOS Genet. 15, e1007997 (2019). 30845139 10.1371/journal.pgen.1007997 PMC6424463

[17] Y. Hirakawa, T. Fujimoto, S. Ishida, N. Uchida, S. Sawa, T. Kiyosue, K. Ishizaki, R. Nishihama, T. Kohchi, J. L. Bowman, Induction of multichotomous branching by CLAVATA peptide in *Marchantia polymorpha*. Curr. Biol. 30, 3833–3840.e4 (2020). 32822612 10.1016/j.cub.2020.07.016

[18] Y. Hata, J. Kyozuka, Fundamental mechanisms of the stem cell regulation in land plants: Lesson from shoot apical cells in bryophytes. Plant Mol. Biol. 107, 213–225 (2021). 33609252 10.1007/s11103-021-01126-y PMC8648652

[19] J. Cammarata, C. M. Farfan, M. J. Scanlon, A. Roeder, Cytokinin–CLAVATA cross-talk is an ancient mechanism regulating shoot meristem homeostasis in land plants. Proc. Natl. Acad. Sci. U.S.A. 119, e2116860119 (2022). 35344421 10.1073/pnas.2116860119 PMC9168927

[20] O. Olsen, P. Perroud, W. Johansen, V. Demko, DEK1; missing piece in puzzle of plant development. Trends Plant Sci. 20, 70–71 (2015). 25612461 10.1016/j.tplants.2015.01.003

[21] R. Kofuji, M. Hasebe, Eight types of stem cells in the life cycle of the moss *Physcomitrella patens*. Curr. Opin. Plant Biol. 17, 13–21 (2014). 24507489 10.1016/j.pbi.2013.10.007

[22] S. Naramoto, Y. Hata, T. Fujita, J. Kyozuka, The bryophytes *Physcomitrium patens and Marchantia polymorpha* as model systems for studying evolutionary cell and developmental biology in plants. Plant Cell 34, 228–246 (2022). 34459922 10.1093/plcell/koab218 PMC8773975

[23] C. J. Harrison, A. Roeder, E. M. Meyerowitz, J. A. Langdale, Local cues and asymmetric cell divisions underpin body plan transitions in the moss *Physcomitrella patens*. Curr. Biol. 19, 461–471 (2009). 19303301 10.1016/j.cub.2009.02.050

[24] S. Naramoto, Y. Hata, J. Kyozuka, The origin and evolution of the ALOG proteins, members of a plant-specific transcription factor family, in land plants. J. Plant Res. 133, 323–329 (2020). 32052256 10.1007/s10265-020-01171-6

[25] A. Yoshida, T. Suzaki, W. Tanaka, H. Hirano, The homeotic gene *long sterile lemma* (*G1*) specifies sterile lemma identity in the rice spikelet. Proc. Natl. Acad. Sci. U.S.A. 106, 20103–20108 (2009). 19901325 10.1073/pnas.0907896106 PMC2775035

[26] A. Yoshida, M. Sasao, N. Yasuno, K. Takagi, Y. Daimon, R. Chen, R. Yamazaki, H. Tokunaga, Y. Kitaguchi, Y. Satō, Y. Nagamura, T. Ushijima, T. Kumamaru, S. Iida, M. Maekawa, J. Kyozuka, *TAWAWA1*, a regulator of rice inflorescence architecture, functions through the suppression of meristem phase transition. Proc. Natl. Acad. Sci. U.S.A. 110, 767–772 (2012). 23267064 10.1073/pnas.1216151110 PMC3545824

[27] E. Cho, P. Zambryski, *ORGAN BOUNDARY1* defines a gene expressed at the junction between the shoot apical meristem and lateral organs. Proc. Natl. Acad. Sci. U.S.A. 108, 2154–2159 (2011). 21245300 10.1073/pnas.1018542108 PMC3033305

[28] S. Takeda, K. Hanano, A. Kariya, S. Shimizu, L. Zhao, M. Matsui, M. Tasaka, M. Aida, CUP-SHAPED COTYLEDON1 transcription factor activates the expression of *LSH4* and *LSH3*, two members of the ALOG gene family, in shoot organ boundary cells. Plant J. 66, 1066–1077 (2011). 21435050 10.1111/j.1365-313X.2011.04571.x

[29] C. A. MacAlister, S. J. Park, K. Jiang, F. Marcel, A. Bendahmane, Y. Izkovich, Y. Eshed, Z. B. Lippman, Synchronization of the flowering transition by the tomato *TERMINATING FLOWER* gene. Nat. Genet. 44, 1393–1398 (2012). 23143603 10.1038/ng.2465

[30] S. Naramoto, V. A. S. Jones, N. Trozzi, M. Sato, K. Toyooka, M. Shimamura, S. Ishida, K. Nishitani, K. Ishizaki, R. Nishihama, T. Kohchi, L. Dolan, J. Kyozuka, A conserved regulatory mechanism mediates the convergent evolution of plant shoot lateral organs. PLOS Biol. 17, e3000560 (2019). 31815938 10.1371/journal.pbio.3000560 PMC6901180

[31] R. Reski, W. O. Abel, Induction of budding on chloronemata and caulonemata of the moss, *Physcomitrella patens*, using isopentenyladenine. Planta 165, 354–358 (1985). 24241140 10.1007/BF00392232

[32] A. P. Schulz, R. Reski, R. Maldiney, M. Laloue, K. Von Schwartzenberg, Kinetics of cytokinin production and bud formation in *Physcomitrella*: Analysis of wild type, a developmental mutant and two of its *ipt* transgenics. J. Plant Physiol. 156, 768–774 (2000).

[33] E. Steiner, A. Israeli, R. Gupta, I. Shwartz, I. Nir, M. Leibman-Markus, L. Tal, M. Farber, Z. Amsalem, N. Ori, B. Müller, M. Bar, Characterization of the cytokinin sensor *TCSv2* in arabidopsis and tomato. Plant Methods 16, 152 (2020). 33292327 10.1186/s13007-020-00694-2 PMC7670716

[34] T. Kurakawa, N. Ueda, M. Maekawa, K. Kobayashi, M. Kojima, Y. Nagato, H. Sakakibara, J. Kyozuka, Direct control of shoot meristem activity by a cytokinin-activating enzyme. Nature 445, 652–655 (2007). 17287810 10.1038/nature05504

[35] L. Chen, G. B. Jameson, Y. Guo, J. Song, P. E. Jameson, The *LONELY GUY* gene family: From mosses to wheat, the key to the formation of active cytokinins in plants. Plant Biotechnol. J. 20, 625–645 (2022). 35108444 10.1111/pbi.13783 PMC8989509

[36] M. H. Frank, M. J. Scanlon, Cell-specific transcriptomic analyses of three-dimensional shoot development in the moss *Physcomitrella patens*. Plant J. 83, 743–751 (2015). 26123849 10.1111/tpj.12928

[37] M. H. Frank, M. J. Scanlon, Transcriptomic evidence for the evolution of shoot meristem function in sporophyte-dominant land plants through concerted selection of ancestral gametophytic and sporophytic genetic programs. Mol. Biol. Evol. 32, 3033 (2015). 26416978 10.1093/molbev/msv182

[38] W. Lin, Y. Wang, Y. Coudert, D. Kierzkowski, Leaf morphogenesis: Insights from the moss *Physcomitrium patens*. Front. Plant Sci. 12, 736212 (2021). 34630486 10.3389/fpls.2021.736212 PMC8494982

[39] K. Sakakibara, T. Nishiyama, N. Sumikawa, R. Kofuji, T. Murata, M. Hasebe, Involvement of auxin and a homeodomain-leucine zipper I gene in rhizoid development of the moss *Physcomitrella patens*. Development 130, 4835–4846 (2003). 12917289 10.1242/dev.00644

[40] K. Hiratsu, K. Matsui, T. Koyama, M. Ohme-Takagi, Dominant repression of target genes by chimeric repressors that include the EAR motif, a repression domain, in *Arabidopsis*. Plant J. 34, 733–739 (2003). 12787253 10.1046/j.1365-313x.2003.01759.x

[41] M. Kubo, A. Imai, T. Nishiyama, M. Ishikawa, Y. Sato, T. Kurata, Y. Hiwatashi, R. Reski, M. Hasebe, System for stable β-estradiol-inducible gene expression in the moss *Physcomitrella patens*. PLOS ONE 8, e77356 (2013). 24086772 10.1371/journal.pone.0077356 PMC3785464

[42] X. Cao, S. J. Park, J. Van Eck, Z. B. Lippman, Control of inflorescence architecture in tomato by BTB/POZ transcriptional regulators. Genes Dev. 30, 2048–2061 (2016). 27798848 10.1101/gad.288415.116 PMC5066612

[43] P. Peng, L. Liu, J. Fang, J. Zhao, S. Yuan, X. Li, The rice TRIANGULAR HULL1 protein acts as a transcriptional repressor in regulating lateral development of spikelet. Sci. Rep. 7, 13712 (2017). 29057928 10.1038/s41598-017-14146-w PMC5651839

[44] M. S. V. Phan, I. Keren, P. T. Tran, M. Lapidot, V. Citovsky, Arabidopsis LSH10 transcription factor and OTLD1 histone deubiquitinase interact and transcriptionally regulate the same target genes. Comm. Biol. 6, 58 (2023). 10.1038/s42003-023-04424-x PMC9845307 36650214

[45] M. Ishikawa, M. Morishita, Y. Higuchi, S. Ichikawa, T. Ishikawa, T. Nishiyama, Y. Kabeya, Y. Hiwatashi, T. Kurata, M. Kubo, S. Shigenobu, Y. Tamada, Y. Sato, M. Hasebe, Physcomitrella STEMIN transcription factor induces stem cell formation with epigenetic reprogramming. Nat. Plants 5, 681–690 (2019). 31285563 10.1038/s41477-019-0464-2

[46] T. A. Bennett, M. M. Liu, T. Aoyama, N. M. Bierfreund, M. Braun, Y. Coudert, R. J. Dennis, D. O’Connor, X. Y. Wang, C. D. White, E. L. Decker, R. Reski, C. J. Harrison, Plasma membrane-targeted PIN proteins drive shoot development in a moss. Curr. Biol. 24, 2776–2785 (2014). 25448003 10.1016/j.cub.2014.09.054 PMC4251699

[47] J. Cammarata, A. H. K. Roeder, M. J. Scanlon, The ratio of auxin to cytokinin controls leaf development and meristem initiation in *Physcomitrium patens*. J. Exp. Bot. 74, 6541–6550 (2023). 37498739 10.1093/jxb/erad299

[48] V. Chickarmane, S. P. Gordon, P. T. Tarr, M. G. Heisler, E. M. Meyerowitz, Cytokinin signaling as a positional cue for patterning the apical–basal axis of the growing *Arabidopsis* shoot meristem. Proc. Natl. Acad. Sci. U.S.A. 109, 4002–4007 (2012). 22345559 10.1073/pnas.1200636109 PMC3309735

[49] T. Laux, K. Mayer, J. Berger, G. Jürgens, The *WUSCHEL* gene is required for shoot and floral meristem integrity in *Arabidopsis*. Development 122, 87–96 (1996). 8565856 10.1242/dev.122.1.87

[50] Y. Coudert, O. Novák, C. J. Harrison, A KNOX-cytokinin regulatory module predates the origin of indeterminate vascular plants. Curr. Biol. 29, 2743–2750.e5 (2019). 31378615 10.1016/j.cub.2019.06.083

[51] K. Sakakibara, P. Reisewitz, T. Aoyama, T. Friedrich, S. Ando, Y. Sato, Y. Tamada, T. Nishiyama, Y. Hiwatashi, T. Kurata, M. Ishikawa, H. Deguchi, S. A. Rensing, W. Werr, T. Murata, M. Hasebe, T. Laux, *WOX13*-*like* genes are required for reprogramming of leaf and protoplast cells into stem cells in the moss *Physcomitrella patens*. Development 141, 1660–1670 (2014). 24715456 10.1242/dev.097444

[52] C. E. Youngstrom, K. A. Withers, E. E. Irish, C. Cheng, Vascular function of the T3/modern clade *WUSCHEL-Related HOMEOBOX* transcription factor genes predate apical meristem-maintenance function. BMC Plant Biol. 22, 210 (2022). 35462532 10.1186/s12870-022-03590-0 PMC9036803

[53] C.-C. Wu, F. Li, E. M. Kramer, Large-scale phylogenomic analysis suggests three ancient superclades of the WUSCHEL-RELATED HOMEOBOX transcription factor family in plants. PLOS ONE 14, e0223521 (2019). 31603924 10.1371/journal.pone.0223521 PMC6788696

[54] K. Sakakibara, T. Nishiyama, H. Deguchi, M. Hasebe, Class 1 KNOX genes are not involved in shoot development in the moss *Physcomitrella patens* but do function in sporophyte development. Evol. Dev. 10, 555–566 (2008). 18803774 10.1111/j.1525-142X.2008.00271.x

[55] N. W. Ashton, D. J. Cove, The isolation and preliminary characterisation of auxotrophic and analogue resistant mutants of the moss, *Physcomitrella patens*. Mol. Genet. Genom. 154, 87–95 (1977).

[56] T. Nishiyama, Y. Hiwatashi, K. Sakakibara, M. Kato, M. Hasebe, Tagged mutagenesis and gene-trap in the moss, *Physcomitrella patens* by shuttle mutagenesis. DNA Res. 7, 9–17 (2000). 10718194 10.1093/dnares/7.1.9

[57] M. Ishikawa, T. Murata, Y. Sato, T. Nishiyama, Y. Hiwatashi, A. Imai, M. Kimura, N. Sugimoto, A. Akita, Y. Oguri, W. E. Friedman, M. Hasebe, M. Kubo, *Physcomitrella* cyclin-dependent kinase a links cell cycle reactivation to other cellular changes during reprogramming of leaf cells. Plant Cell 23, 2924–2938 (2011). 21862705 10.1105/tpc.111.088005 PMC3180801

[58] Y. Zhang, U. Werling, W. Edelmann, SLiCE: A novel bacterial cell extract-based DNA cloning method. Nucleic Acids Res. 40, e55 (2012). 22241772 10.1093/nar/gkr1288 PMC3333860

