# Supplementary material for: Cytokinin and ALOG proteins regulate pluripotent stem cell identity in the moss Physcomitrium patens
Source: Sci Adv. 2024 Aug 28;10(35):eadq6082. doi: 10.1126/sciadv.adq6082 (PMC11352904; doi:10.1126/sciadv.adq6082)
Supplement: Supplementary file 1 — Figs. S1 to S7 Table S1 Legends for movies S1 to S4 Legend for data S1 [file sciadv.adq6082_sm.pdf]

Science Advances
AAAS

Supplementary Materials for

Cytokinin and ALOG proteins regulate pluripotent stem cell identity in the moss *Physcomitrium patens*

Yuki Hata *et al.*

Corresponding author: Junko Kyozuka, junko.kyozuka.e4@tohoku.ac.jp

Sci. Adv. **10**, eadq6082 (2024)
DOI: 10.1126/sciadv.adq6082

The PDF file includes:

Figs. S1 to S7
Table S1
Legends for movies S1 to S4
Legend for data S1

Other Supplementary Material for this manuscript includes the following:

Movies S1 to S4
Data S1

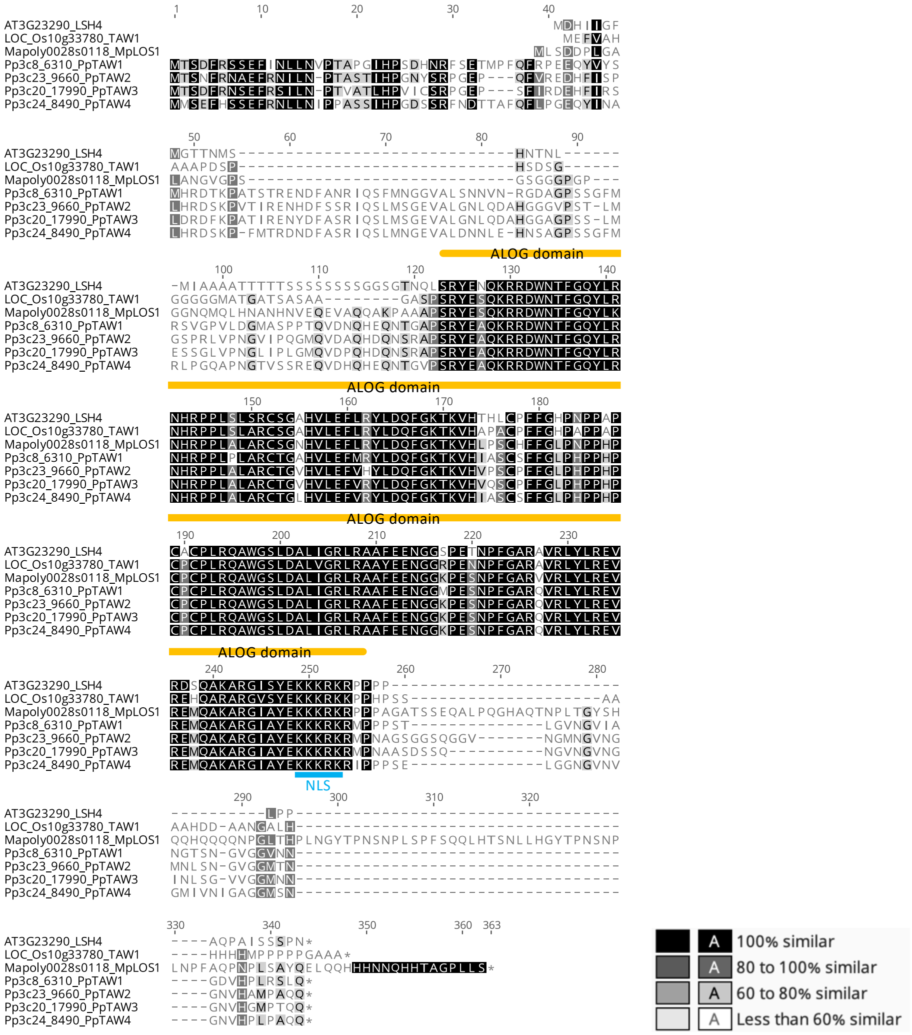

Fig. S1. Conservation of amino acid sequences in ALOG proteins.

Alignment of amino acid sequences of ALOG proteins. Species shown in the alignment are *Physcomitrium patens* (Pp), *Marchantia polymorpha* (Mapoly), *Arabidopsis thaliana* (AT), and *Oryza sativa* (Os). Each amino acid letters are highlighted by black colors based on similarity. Region of the ALOG domain is indicated by orange bars. Position of the nuclear localization signal (NLS) is shown by a cyan bar.

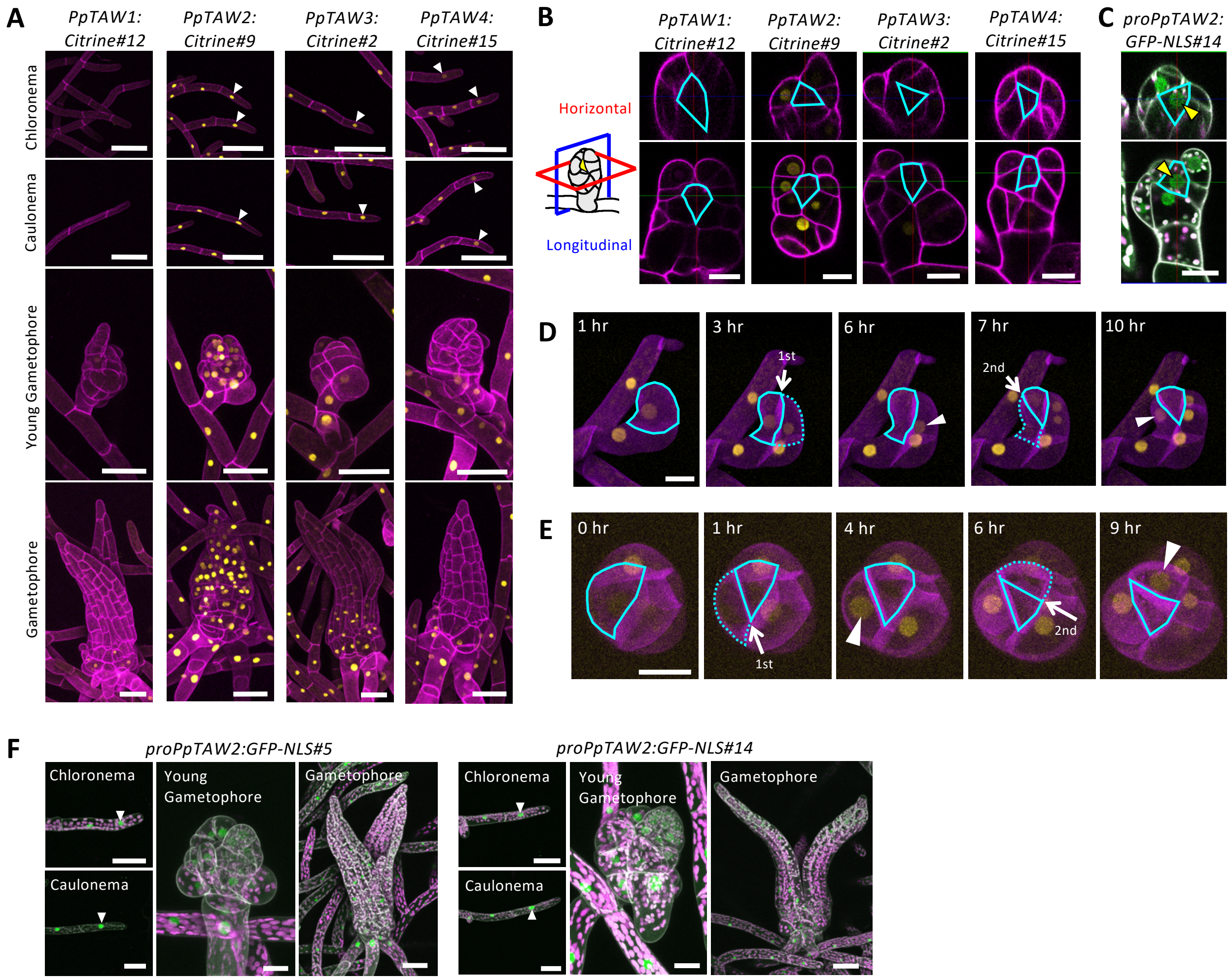

Fig. S2. Localization of PpTAW proteins and promoter activity of *PpTAW2* in other lines.

(**A**) Localizations of PpTAW:Citrine fluorescence (yellow) in protonema, young gametophore and gametophore. PpTAW:Citrine fluorescence is visible in the chloronemal and caulonemal apical cells (white arrowheads). Cell walls were stained with propidium iodide (magenta). (**B**) Localizations of PpTAW:Citrine fluorescence (yellow) in the shoot apical meristem (SAM). The top panels show horizontal (red square) views, and the bottom panels show longitudinal (blue square) views. Cell walls are stained with propidium iodide (magenta). The outlines of the gametophore apical cell is marked with cyan lines. (**C**) Promoter activity of *PpTAW2*. GFP fluorescence (green) driven by the *PpTAW2* promoter is shown. Cell walls are stained with propidium iodide (white). The magenta color represents the autofluorescence of chloroplasts. Yellow arrowheads indicate the GFP fluorescence in the gametophore apical cell. (**D** and **E**) Time-lapse imaging of PpTAW2:Citrine (yellow) localization during the division of gametophore apical cells in an initiating gametophore (D) and a growing young gametophore (E). Lateral view (D) and top view (E) of growing gametophores are shown. LTI6b:RFP (magenta) was simultaneously imaged for the visualization of cell outlines. Solid and dashed cyan lines show outlines of the gametophore apical cell and newly formed merophyte, respectively. White arrows indicate the cell division planes formed during the observation time. White arrowheads indicate the PpTAW2:Citrine signal that appeared in the merophyte after the division of the gametophore

apical cell. No PpTAW2:Citrine signal is observed in the gametophore apical cell. (**F**) Promoter activity of PpTAW2 (green) during developmental processes from protonema to gametophores. Cell walls were stained with propidium iodide (white). Magenta color shows autofluorescence of chloroplasts. White arrowheads represent promoter activity in the chloromena and caulonema stem cell. Scale bars, 50 μm (A, F, Chloronema, Caulonema, and Gametophore), and 20 μm (B to E, and F, Young Gametophore).

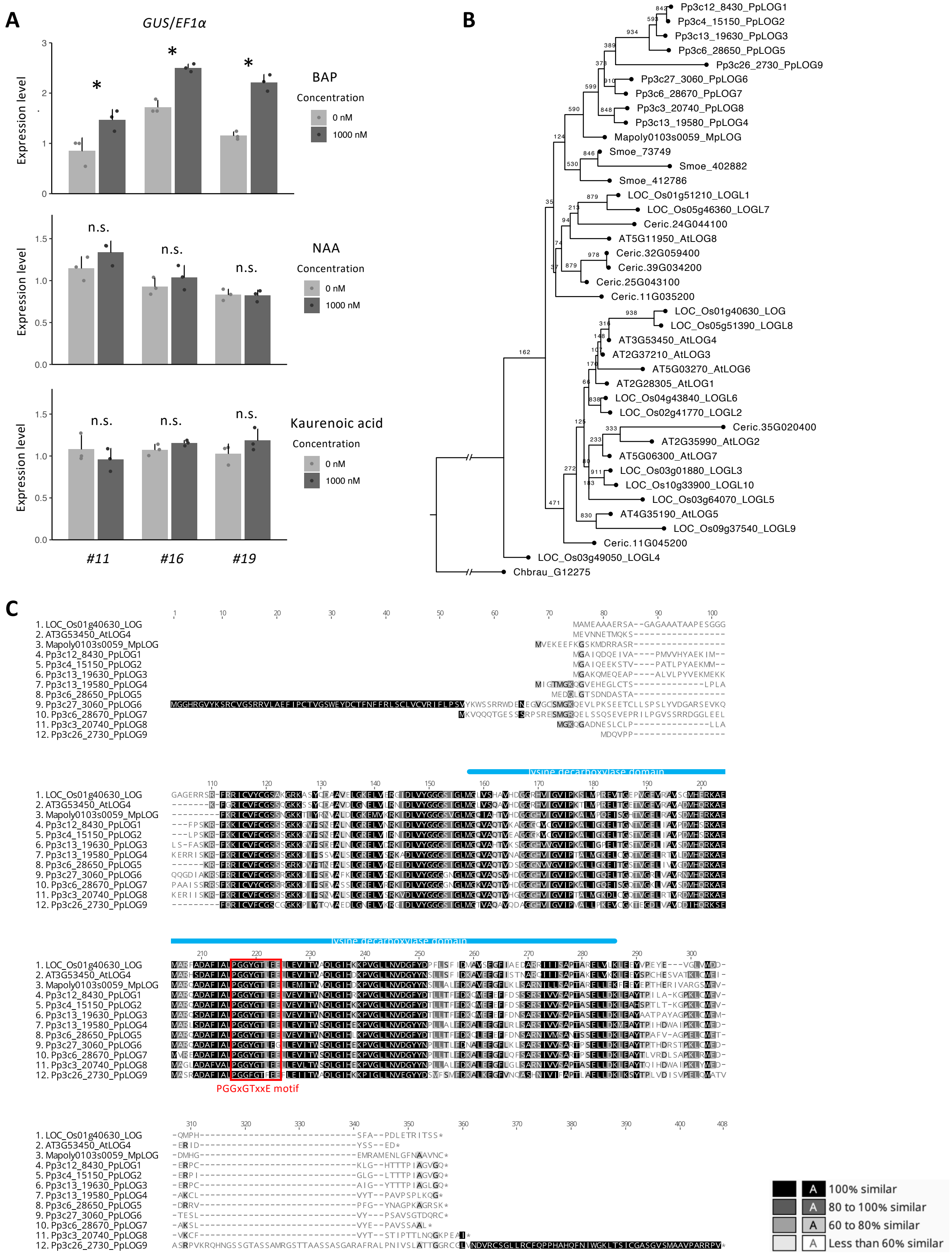
A
GUS/EF1α
Expression level
BAP
Concentration
0 nM
1000 nM
NAA
n.s.
Kaurenoic acid
#11
#16
#19
B
Pp3c12_8430_PpLOG1
Pp3c4_15150_PpLOG2
Pp3c13_19630_PpLOG3
Pp3c6_28650_PpLOG5
Pp3c26_2730_PpLOG9
Pp3c27_3060_PpLOG6
Pp3c6_28670_PpLOG7
Pp3c3_20740_PpLOG8
Pp3c13_19580_PpLOG4
Mapoly0103s0059_MpLOG
Smoe_73749
Smoe_402882
Smoe_412786
LOC_Os01g51210_LOGL1
LOC_Os05g46360_LOGL7
Ceric.24G044100
AT5G11950_AtLOG8
Ceric.32G059400
Ceric.39G034200
Ceric.25G043100
Ceric.11G035200
LOC_Os01g40630_LOG
LOC_Os05g51390_LOGL8
AT3G53450_AtLOG4
AT2G37210_AtLOG3
AT5G03270_AtLOG6
AT2G28305_AtLOG1
LOC_Os04g43840_LOGL6
LOC_Os02g41770_LOGL2
Ceric.35G020400
AT2G35990_AtLOG2
AT5G06300_AtLOG7
LOC_Os03g01880_LOGL3
LOC_Os10g33900_LOGL10
LOC_Os03g64070_LOGL5
AT4G35190_AtLOG5
LOC_Os09g37540_LOGL9
Ceric.11G045200
LOC_Os03g49050_LOGL4
Chbrau_G12275
C
1. LOC_Os01g40630_LOG
2. AT3G53450_AtLOG4
3. Mapoly0103s0059_MpLOG
4. Pp3c12_8430_PpLOG1
5. Pp3c4_15150_PpLOG2
6. Pp3c13_19630_PpLOG3
7. Pp3c13_19580_PpLOG4
8. Pp3c6_28650_PpLOG5
9. Pp3c27_3060_PpLOG6
10. Pp3c6_28670_PpLOG7
11. Pp3c3_20740_PpLOG8
12. Pp3c26_2730_PpLOG9
lysine decarboxylase domain
PGGxGTxxE motif
100% similar
80 to 100% similar
60 to 80% similar
Less than 60% similar

Fig. S3. Validation of TCSv2 reporter in *P. patens* and LOG protein family in land plants.

(**A**) Response of *TCSv2:GUS* expression to the cytokinin (BAP), auxin (NAA), and kaurenoic acid measured by qRT-PCR. Three independent lines (#11, #16, and #19) were examined. The phytohormone treatment was conducted for 3 hours. Statistical significance was evaluated by student's t-test ($n = 3$, $p < 0.05$). (**B**) Phylogenetic tree of LOG proteins in land plants. Species shown in the tree are *Chara braunii* (Chbrau), *Physcomitrium patens* (Pp), *Marchantia polymorpha* (Mapoly), *Selaginella moellendorffii* (Sm), *Ceratopteris richardii* (Ceric), *Arabidopsis thaliana* (AT), and *Oryza sativa* (Os). A LOG protein in *Chara braunii* (Chbrau_G29454) was included as an outgroup. The bootstrap value is indicated beside the branch point. (**C**) Alignment of amino acid sequences of LOG proteins. Each amino acid letters are highlighted by black colors based on similarity. Region of the lysine decarboxylase domain is indicated by blue bars. Position of the conserved PGGxGTxxE motif is shown by a red box.

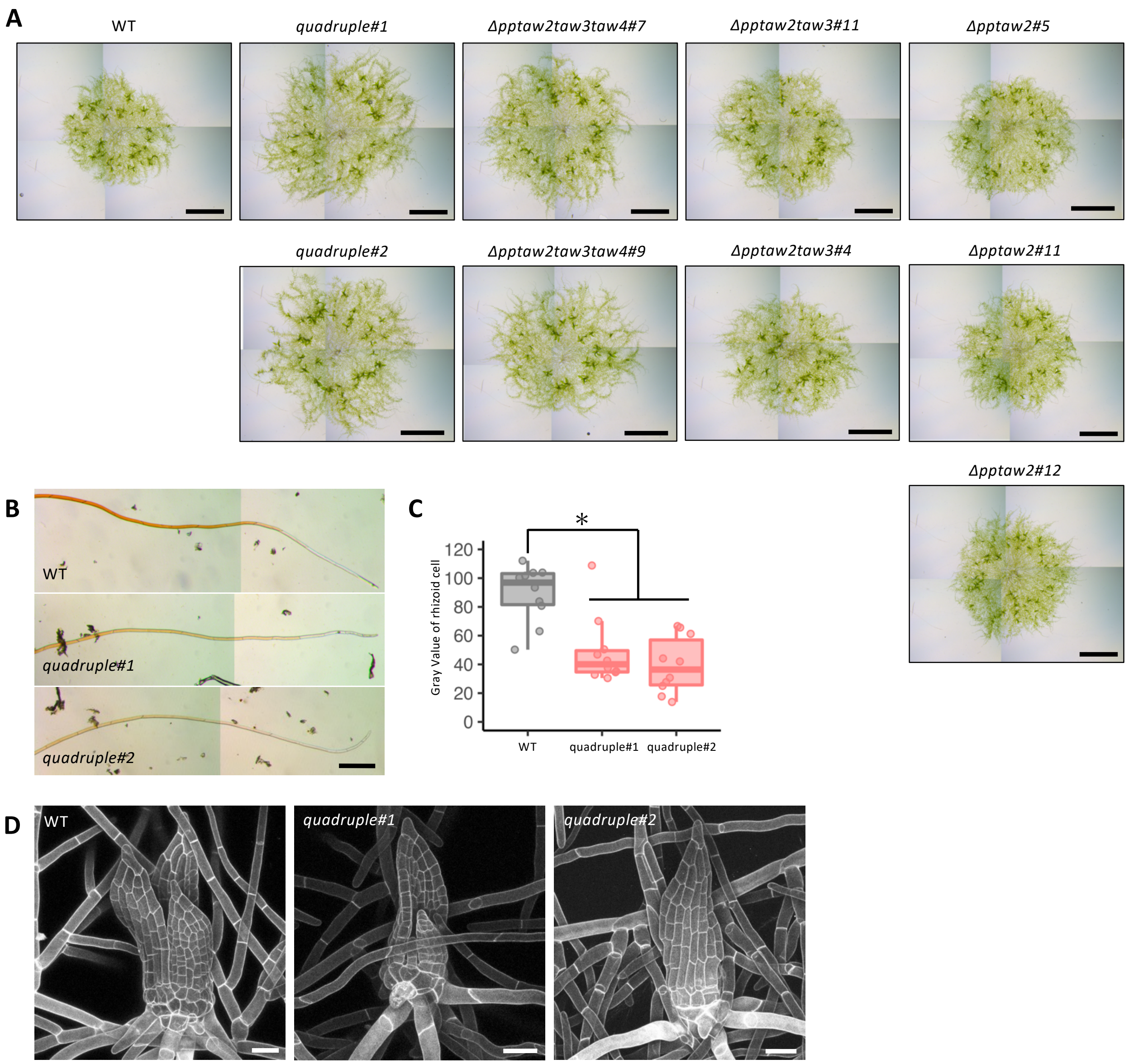

Fig. S4. Phenotype of *PpTAWs* loss-of-function mutants.

(**A**) 23 days plant colony of WT and the *PpTAWs* loss-of-function mutants. (**B**) Pigmentation of rhizoids in the WT and quadruple mutants. (**C**) Gray value of the 10th rhizoid cell from tip in the in the WT and quadruple mutants. Statistical significance was assessed by student's t-test ($n = 10$, $p < 0.05$). (**D**) Confocal images of gametophores in WT and quadruple mutants. Scale bars, 4 mm (A), 200 μm (B), and 50 μm (D).

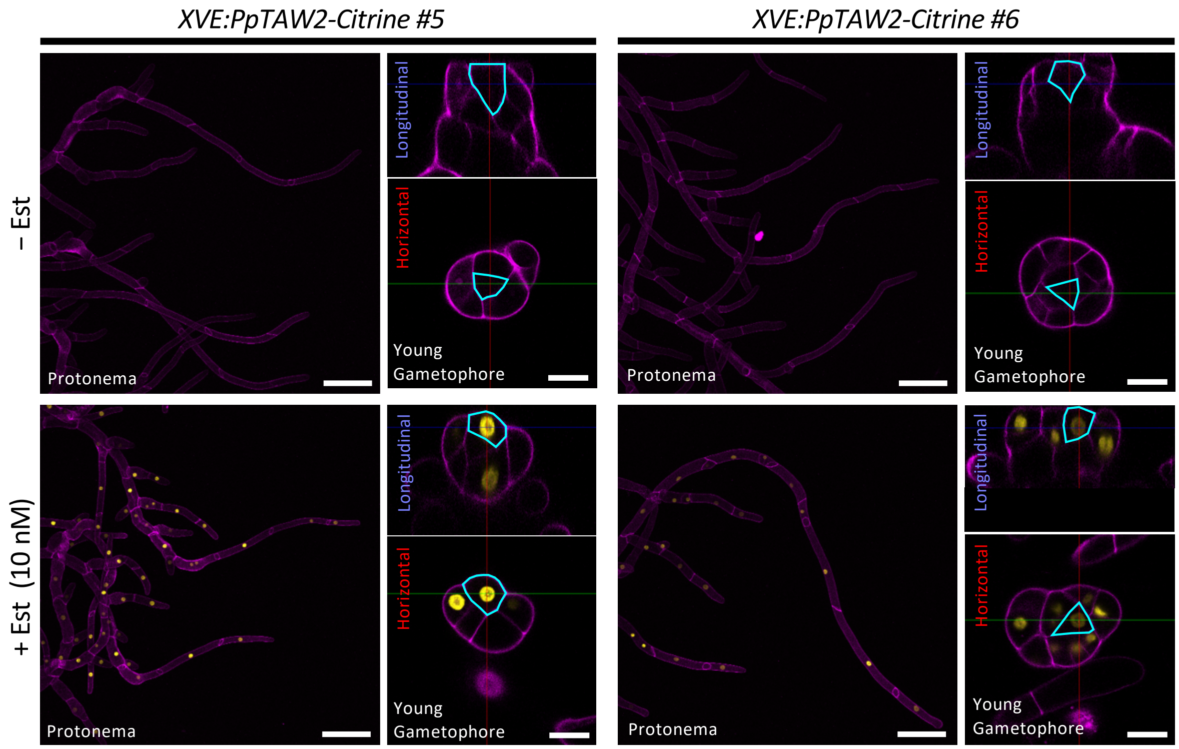

Fig. S5. Ectopic localization of PpTAW2 protein.

The β-estradiol inducible system of PpTAW2-Citrine. β-estradiol treatment (10 nM, 24 hours) induced the expression of the PpTAW2-Citrine (yellow) localized to nuclei in both protonema and young gametophores including the gametophore apical cell indicated by cyan lines. No signal was detected in the control. Cell walls were stained with propidium iodide staining (magenta). Scale bars, 100 µm in protonema images, and 20 µm in young gametophore images.

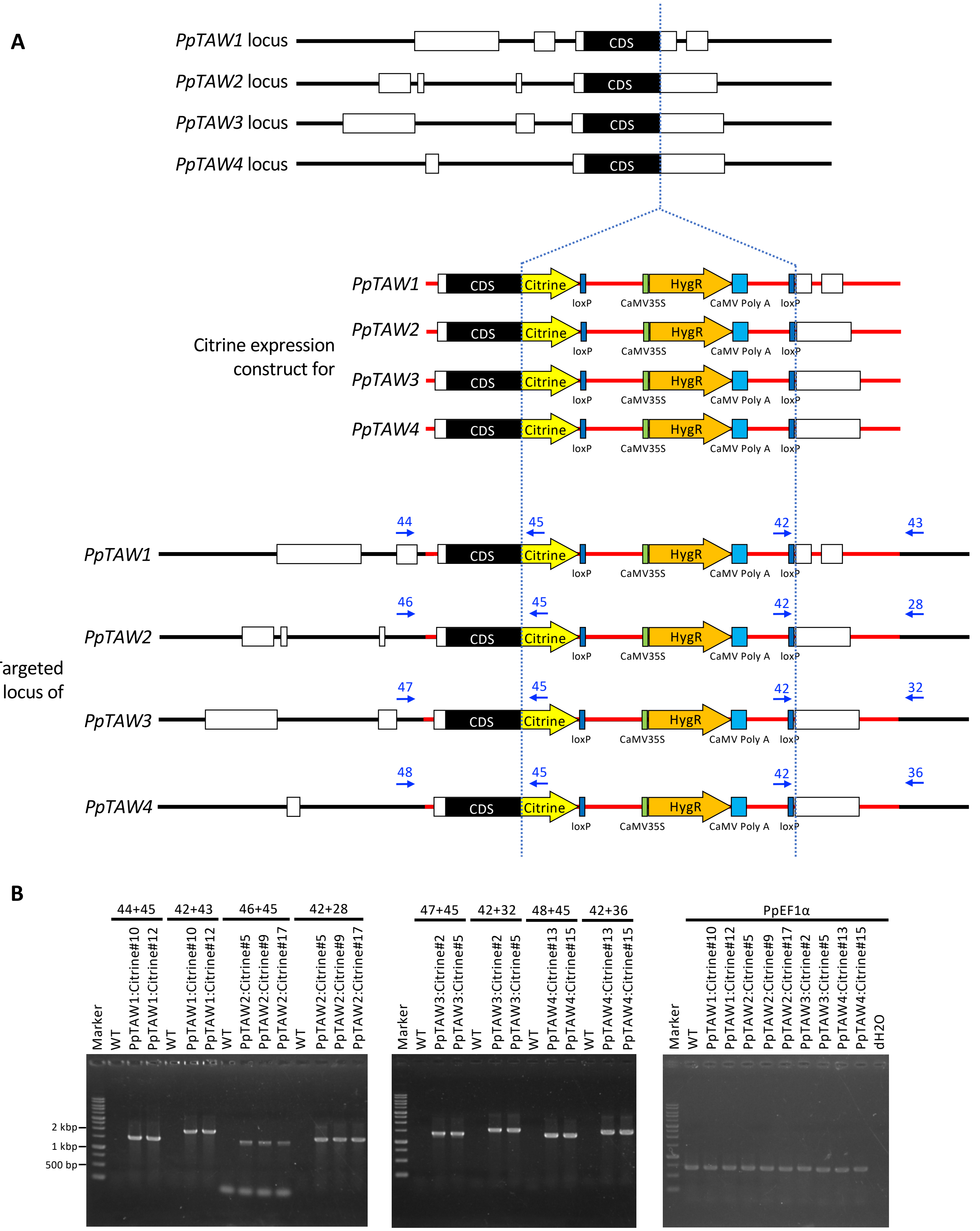

Fig. S6. Construction for visualization of PpTAW1-4 localization by Knocking in Citrine.

(**A**) Schematic diagrams of *PpTAW1-4* loci and targeting of the constructs. White and black boxes indicate exons and protein- coding sequences (CDS), respectively. Blue arrows with a number

show the position of primers used in genotyping. The numbers are corresponding to the primer numbers in Table S1. (**B**) Examination of PCR products for the genotyping by the agarose gel electrophoresis. Numbers of the top show primer pairs used in the PCR. The numbers are corresponding to the primer numbers in Table S1. Genomic region encoding *PpEF1α* was amplified simultaneously as a positive control of the PCR (primer #53 and #54 were used).

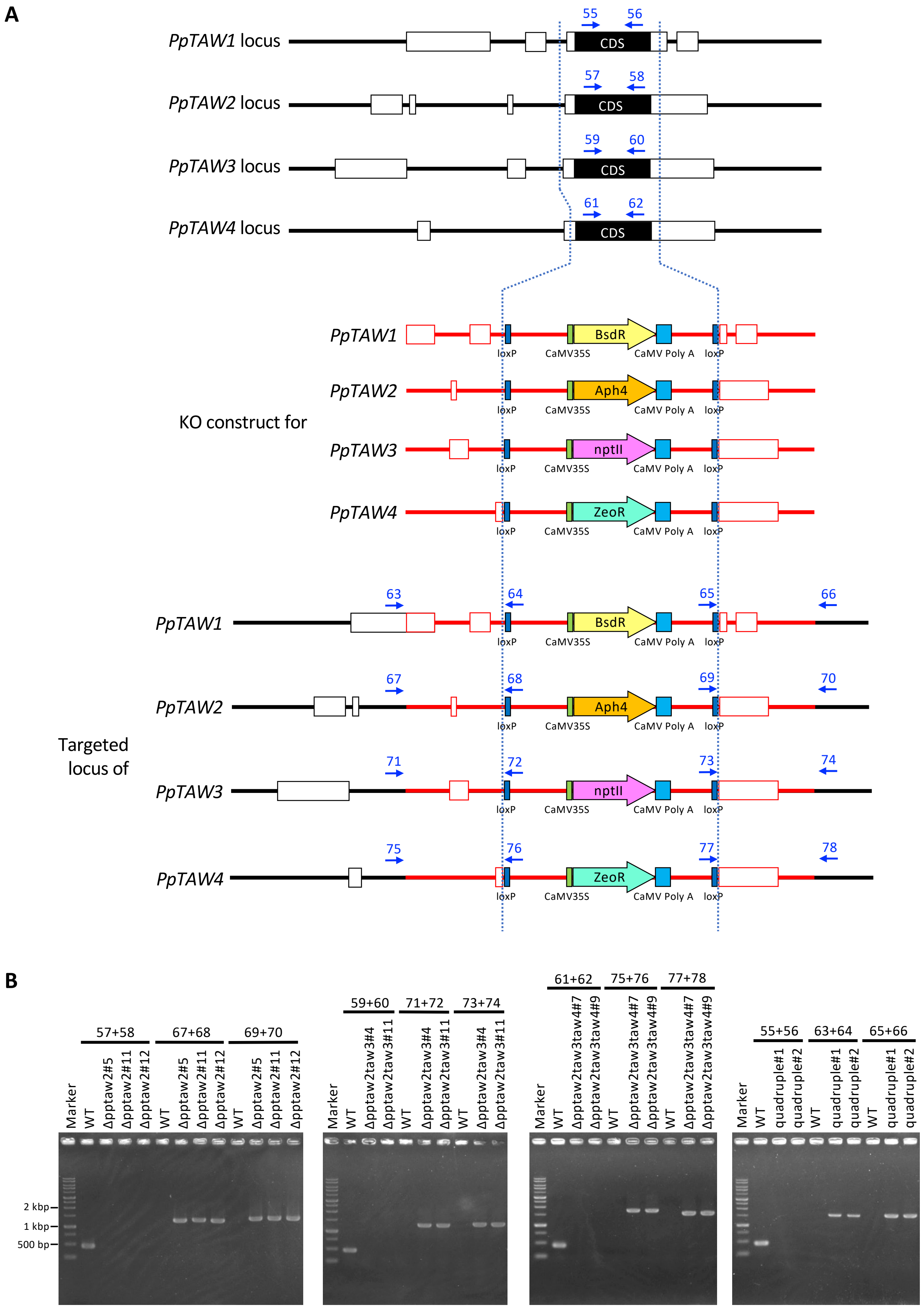
A
PpTAW1 locus
PpTAW2 locus
PpTAW3 locus
PpTAW4 locus
CDS
55
56
57
58
59
60
61
62
KO construct for
PpTAW1
PpTAW2
PpTAW3
PpTAW4
BsdR
Aph4
nptII
ZeoR
loxP
CaMV35S
CaMV Poly A
Targeted locus of
63
64
65
66
67
68
69
70
71
72
73
74
75
76
77
78
B
57+58
67+68
69+70
59+60
71+72
73+74
61+62
75+76
77+78
55+56
63+64
65+66
Marker
WT
Δpptaw2#5
Δpptaw2#11
Δpptaw2#12
Δpptaw2taw3#4
Δpptaw2taw3#11
Δpptaw2taw3taw4#7
Δpptaw2taw3taw4#9
quadruple#1
quadruple#2
2 kbp
1 kbp
500 bp

Fig. S7. Construction for *PpTAW1-4* KO lines.

(**A**) Schematic diagrams of *PpTAW1-4* loci and targeting of the constructs. White and black boxes indicate exons and protein- coding sequences (CDS), respectively. Blue arrows with a number show the position of primers used in genotyping. The numbers are corresponding to the primer numbers in Table S1. (**B**) Examination of PCR products for the genotyping by the agarose gel electrophoresis. Numbers of the top show primer pairs used in the PCR. The numbers are corresponding to the primer numbers in Table S1. Firstly, *Δpptaw2* single mutants were obtained and *Δpptaw2Δpptaw3* double mutants were generated from *Δpptaw2#5. Δpptaw2Δpptaw3Δpptaw4* triple mutants were generated from *Δpptaw2Δpptaw3#11.* Finally, quadruple mutants were generated from *Δpptaw2Δpptaw3Δpptaw4#9*.

Table S1. Primers used in this study

#	primer name	sequence (5'→3')	description
1	HRCit5'TAW1clo_F	ATCGTATGCATTTATATAACCATT	for construction of PpTAW1:Citrine
2	HRCit5'TAW1clo_R	TGATCCTCCTCCCTGCAGTGACCGGAGAG	for construction of PpTAW1:Citrine
3	HRCit3'TAW1clo_F	TAACAAAGTTTTGACCTCCGAA	for construction of PpTAW1:Citrine
4	HRCit3'TAW1clo_R	GATATCGATTGAAGAGGCGGT	for construction of PpTAW1:Citrine
5	HRCit5'TAW2clo_F	ATCACATTCACATTTGTAGTGC	for construction of PpTAW2:Citrine
6	HRCit5'TAW2clo_R	TGATCCTCCTCCTTGCTGCGCCGGCATGG	for construction of PpTAW2:Citrine
7	HRCit3'TAW2clo_F	GCGAACTGTTTGAACAGAC	for construction of PpTAW2:Citrine
8	HRCit3'TAW2clo_R	GATATCAATTCTTTCCGCTCA	for construction of PpTAW2:Citrine
9	HRCit5'TAW3clo_F	ATCACATTCGCGGAGATA	for construction of PpTAW3:Citrine
10	HRCit5'TAW3clo_R	TGATCCTCCTCCCTGCTGCGTCGGCATGC	for construction of PpTAW3:Citrine
11	HRCit3'TAW3clo_F	GCGAACTGTTTGAACATACG	for construction of PpTAW3:Citrine
12	HRCit3'TAW3clo_R	GATATCGTCGATTGATGCGAA	for construction of PpTAW3:Citrine
13	HRCit5'TAW4clo_F	ATCGATATGTAGCAAACGAC	for construction of PpTAW4:Citrine
14	HRCit5'TAW4clo_R	TGATCCTCCTCCCTGCTGTGCTGGGAGAG	for construction of PpTAW4:Citrine
15	HRCit3'TAW4clo_F	GTACAATGCTCTGATCTGCG	for construction of PpTAW4:Citrine
16	HRCit3'TAW4clo_R	GATATCCAGAAGGCAGAAGCA	for construction of PpTAW4:Citrine
17	proPpTAW2_clo_F	AGGATCCCCCCCCCCTGGAAGGGCACAAGGAACTG	cloning PpTAW2 promoter
18	proPpTAW2_clo_R	TGGATCAGCCATCCCCCAAAGATACTGGCTCAGCGA	cloning PpTAW2 promoter
19	M13F	TGTAAAACGACGGCCAGT	amplification of TCSv2
20	TCSv2_in_3xVENUS_reverse	CGATTTCGAACCCGGGGTAC	amplification of TCSv2
21	PpTAW1_5'UTR_CloF	GTCTTGCCGATGCTGGTTTG	for construction of PpTAW1 KO
22	PpTAW1_5'UTR_CloR	GAAAGAAACACTGCGCACCA	for construction of PpTAW1 KO
23	PpTAW1_3'UTR_CloF	GCTTCCATGCGCAAAGGAAA	for construction of PpTAW1 KO
24	PpTAW1_3'UTR_CloR	CTTCTTCACGGGCAGGACTC	for construction of PpTAW1 KO
25	PpTAW2_5'UTR_CloF	TTTAGCCCTGCACTCGTTGT	for construction of PpTAW2 KO
26	PpTAW2_5'UTR_CloR	GCAGGGGGTATACGATGCTC	for construction of PpTAW2 KO
27	PpTAW2_3'UTR_CloF	GTCACAGTCCCGTCACAGTT	for construction of PpTAW2 KO
28	PpTAW2_3'UTR_CloR	TGTCTTCTGCTTGCCTTGCT	for construction of PpTAW2 KO
29	PpTAW3_5'UTR_CloF	TGCGTTAGCTTGGAACCTGT	for construction of PpTAW3 KO
30	PpTAW3_5'UTR_CloR	GCAGGAAGCCGAGCTCATAA	for construction of PpTAW3 KO
31	PpTAW3_3'UTR_CloF	CACAAAGACGACGAGGACGA	for construction of PpTAW3 KO
32	PpTAW3_3'UTR_CloR	GCGTATGAGTATGGCAGGCT	for construction of PpTAW3 KO
33	PpTAW4_5'UTR_CloF	ACTGTTTCAGCCCTCACGAG	for construction of PpTAW4 KO
34	PpTAW4_5'UTR_CloR	CGTAGCGAAGAAGCACTTGC	for construction of PpTAW4 KO
35	PpTAW4_3'UTR_CloF	TCTGATCTGCGAAACAGCGA	for construction of PpTAW4 KO
36	PpTAW4_3'UTR_CloR	GCTCTGCAGACTTCTTCGGA	for construction of PpTAW4 KO
37	topoPpTAW2clo_F	CACCATGACGAGTAACTTTCGCA	cloning PpTAW2 CDS
38	topoPpTAW2clo_R	TCATTGCTGCGCCGGCA	cloning PpTAW2 CDS
39	pDONR_PpTAW2_SRDXintr_F	CTGCGCCTGGGCTTTGCGTGAAAGGGTGGGCGCGC	for SRDX introduction
40	pDONR_PpTAW2_SRDXintr_R	TTCCAGATCCAGATCCAGTTGCTGCGCCGGCATGG	for SRDX introduction
41	Citrine_clo_R	TTACTTGTACAGCTCGTCCATG	amplification of PpTAW2:Citrine
42	HRCitT1geno3_F	AGTTATCCCTCACACCGGTG	targeting check for PpTAW:Citrine lines
43	HRCitT1geno3_R	CGGTGAGTTGGTCTGACACA	targeting check for PpTAW:Citrine lines
44	HRCitT1geno5_F	CCCCGGTTCTGATCGTGTTT	targeting check for PpTAW:Citrine lines
45	HRCitT1geno5_R	TGAACTTGTGGCCGTTTACG	targeting check for PpTAW:Citrine lines
46	CRIT2seq.ck_F_2	GAGCATCGTATACCCCCTGC	targeting check for PpTAW:Citrine lines
47	HRCitT3geno5_F	TTATGAGCTCGGCTTCCTGC	targeting check for PpTAW:Citrine lines
48	CRIT4seq.ck_F_4	GAGTGCTTGTCTTGGCTTCC	targeting check for PpTAW:Citrine lines
49	HR_pPGX8_5'geno_F	TGCAACATTTGGAGTTGGCA	targeting check for promoter:NGG lines
50	HR_pPIG1b_NGGII_5'geno_R	GGGGGATCCACTAGTTCTAGA	targeting check for promoter:NGG lines
51	HR_pPGX8_3'geno_F	ATAATCCGCATAAGCCCCCG	targeting check for promoter:NGG lines
52	HR_pPGX8_3'geno_R	TGCATAGTTTAATTAGCAGAGTTGT	targeting check for promoter:NGG lines
53	semi_qPCR_EF1alpha_F	TGTGGAAGTTCGAGACCGTG	for positive control of PCR
54	semi_qPCR_EF1alpha_R	GCTTGTCAGACGGCCTTTTG	for positive control of PCR
55	CRIT1-2nd-alle.seq.ck_F	CATGAGATCAGTCGGACCGG	deletion check for PpTAW1 KO lines
56	CRIT1-2nd-alle.seq.ck_R	CCTGGCCTTAGCTTGCATCT	deletion check for PpTAW1 KO lines
57	CRIT2-2nd-alle.seq.ck_F	TCAAGCCGGATACAGTCCCT	deletion check for PpTAW2 KO lines
58	CRIT2-2nd-alle.seq.ck_R	CTCTCAGGTTTGCCCCCATT	deletion check for PpTAW2 KO lines
59	CRIT3-2nd-alle.seq.ck_F	AGTGGTGAAGTTGCCCTTGG	deletion check for PpTAW3 KO lines
60	CRIT3-2nd-alle.seq.ck_R	CAAGTACAGACGCACTTGGC	deletion check for PpTAW3 KO lines
61	CRIT4-2nd-alle.seq.ck_F	ACGACTTTGCAAGCCGGATA	deletion check for PpTAW4 KO lines
62	CRIT4-2nd-alle.seq.ck_R	TCAAATGCGGCTCGTAACCT	deletion check for PpTAW4 KO lines
63	HR_KOT1_5'geno_F	GTGCAGAAGTACCAGCTTGT	targeting check for PpTAW1 KO lines
64	HR_KOT1_5'geno_R	GGGGGCTGCAGGAATATAACT	targeting check for PpTAW1 KO lines
65	HR_KOT1_3'geno_F	AGTTATCCCTCACACCGGTG	targeting check for PpTAW1 KO lines
66	HR_KOT1_3'geno_R	TGCTTGCATACTCGGATTCTCT	targeting check for PpTAW1 KO lines
67	HR_KOT2_5geno_F	CGTTGCTTAGAAAACCCGCA	targeting check for PpTAW2 KO lines
68	HR_KOT2_5geno_R	GGGGGCTGCAGGAATATAACT	targeting check for PpTAW2 KO lines
69	HR_KOT2_3geno_F	AGTTATCCCTCACACCGGTG	targeting check for PpTAW2 KO lines
70	HR_KOT2_3geno_R	CATAGTTGGCGATGTGTGCG	targeting check for PpTAW2 KO lines
71	HR_KOT3_pTN182_5'geno_F	TGGCTGTTTCTGTTGGTACCA	targeting check for PpTAW3 KO lines
72	HR_KOT3_pTN182_5'geno_R	TGACATTTTTGGAGTAGGGGG	targeting check for PpTAW3 KO lines
73	HR_KOT3_pTN182_3'geno_F	AGTTATCCCTCACACCGGTG	targeting check for PpTAW3 KO lines
74	HR_KOT3_pTN182_3'geno_R	GTATGCAGATGTACGCGACG	targeting check for PpTAW3 KO lines
75	HRT4geno5'_F	TTTGTGCTCGTTGACGCTTG	targeting check for PpTAW4 KO lines
76	HRT4geno3'_R	CAAAAACGACCTCACCAGCC	targeting check for PpTAW4 KO lines
77	pTN186_SmaI_seq.check.F	AGTTATCCCTCACACCGGTG	targeting check for PpTAW4 KO lines
78	pZeo_5insert_ck_R	AGCCCTTTGGTCTTCTGAGA	targeting check for PpTAW4 KO lines
79	HR_pPGX8_5'geno_F	TGCAACATTTGGAGTTGGCA	targeting check for β-estradiol inducible lines
80	HR_pPGX8_5'geno_R	TGTACAGTACGTCGAGGGGA	targeting check for β-estradiol inducible lines
81	HR_pPGX8_3'geno_F	ATAATCCGCATAAGCCCCCG	targeting check for β-estradiol inducible lines
82	HR_pPGX8_3'geno_R	TGCATAGTTTAATTAGCAGAGTTGT	targeting check for β-estradiol inducible lines
83	qPCRforPpTAW2_No.2_F	TGCACAGGGGTCCATGTGTT	for qRT-PCR of PpTAW2
84	qPCRforPpTAW2_No.2_R	GGTGCGGCAACCCAAAGAAA	for qRT-PCR of PpTAW3
85	qPCRforPpCKX1_F2	GGCGGCACTCTATCGAATGC	for qRT-PCR of PpCKX1
86	qPCRforPpCKX1_R2	GTAGGCGTGCATTGTACCACC	for qRT-PCR of PpCKX1
87	qPCRforPpACT5_F	GTCAACAGCGGATTCCCAGC	for qRT-PCR of PpACT5
88	qPCRforPpACT5_R	ACCCTCTCCGGCCATATTGC	for qRT-PCR of PpACT5
89	GUS_pPCR_2_F	TCTACTTTACTGGCTTTGGTCG	for qRT-PCR of GUS
90	GUS_pPCR_2_R	CGTAAGGGTAATGCGAGGTAC	for qRT-PCR of GUS
91	qPCRforPpEF1a_No2_F	ACGCGTTGTTGGCTTTCACT	for qRT-PCR of PpEF1α
92	qPCRforPpEF1a_No2_R	GCACTGGAGTACTTCGGGGT	for qRT-PCR of PpEF1α

Movie S1.

Time-lapse movie of PpTAW2:Citrine (yellow) localization during the division of gametophore apical cells in an initiating gametophore in Fig. 1I. LTI6b:RFP (magenta) was simultaneously imaged for the visualization of cell outlines. A scale bar, 20 μm.

Movie S2.

Time-lapse movie of PpTAW2:Citrine (yellow) localization during the division of gametophore apical cells in an initiating gametophore in fig. S2D. LTI6b:RFP (magenta) was simultaneously imaged for the visualization of cell outlines. A scale bar, 20 μm.

Movie S3.

Time-lapse movie of PpTAW2:Citrine (yellow) localization during the division of gametophore apical cells in a growing young gametophore in Fig. 1J. LTI6b:RFP (magenta) was simultaneously imaged for the visualization of cell outlines. A scale bar, 20 μm.

Movie S4.

Time-lapse movie of PpTAW2:Citrine (yellow) localization during the division of gametophore apical cells in a growing young gametophore in fig. S2E. LTI6b:RFP (magenta) was simultaneously imaged for the visualization of cell outlines. A scale bar, 20 μm.

Data S1: Source Data

All raw data values are presented in the Source Data.
